# Hybrids as mirrors of the past: genomic footprints reveal spatio-temporal dynamics and extinction risk of alpine extremophytes in the mountains of Central Asia

**DOI:** 10.3389/fpls.2024.1369732

**Published:** 2024-04-17

**Authors:** Anna Wróbel, Ewelina Klichowska, Marcin Nobis

**Affiliations:** ^1^ Institute of Botany, Faculty of Biology, Jagiellonian University, Kraków, Poland; ^2^ Doctoral School of Exact and Natural Sciences, Jagiellonian University, Kraków, Poland

**Keywords:** alpine species, Central Asia, climate change, comparative phylogeography, interspecific hybridization, phylogenetics, population genetics, Puccinellia

## Abstract

Hybridization is one of the key processes shaping lineage diversification, particularly in regions that experienced strong climate oscillations. The alpine biome with its rich history of glacial-interglacial cycles and complex patterns of species distribution shifts offers an excellent system to investigate the impact of gene flow on population dynamics and speciation, important issues for evolutionary biology and biodiversity conservation. In this study, we combined genomic data (DArTseq), chloroplast markers, and morphology to examine phylogenetic relationships and the permeability of species boundaries and their evolutionary outcomes among the alpine extremophilic species of *Puccinellia* (Poaceae) in the Pamir Mountains, a part of the Mountains of Central Asia biodiversity hotspot. We determined the occurrence of interspecific hybrids between *P. himalaica* and *P. pamirica*, which demonstrated almost symmetric ancestry from their parental species and did not show signals of introgression. According to our integrative revision, the natural hybrids between *P. himalaica* and *P. pamirica* should be classified as *Puccinellia* ×*vachanica* (pro species). Using approximate Bayesian computation for population history inference, we uncovered that *P. himalaica* hybridized with *P. pamirica* independently in multiple localities over the Holocene. Hybrids inherited the fine-scale genetic structure from their parental species, which developed these patterns earlier, during the Late Pleistocene. Hybridization had different consequences for the involved parental lineages, likely playing an important role in a continuing decline of *P. himalaica* in the Pamir Mountains over the Holocene. Our results show that *P. himalaica* should be considered a critically endangered species in the Pamir Mountains and could also be retreating across its entire range of distribution in High Mountain Asia. Using a comparative phylogeographic framework, we revealed the risk of extinction of a cold-adapted alpine species in a global biodiversity hotspot. This study highlights that genomics could unravel diversity trends under climate change and provides valuable evidence for conservation management.

## 1 Introduction

Hybridization is one of the key drivers that shape the evolutionary history of Earth’s biodiversity (Mallet, 2005; Soltis and Soltis, 2009; Taylor and Larson, 2019). Reticulate processes can trigger far-reaching and often contrasting consequences in various biological systems (Abbott et al., 2016; Runemark et al., 2019; Nieto Feliner et al., 2023). Interspecific gene flow may promote diversification and adaptive divergence (Edelman et al., 2019; Hodel et al., 2022; Slovák et al., 2023) or, alternatively, homogenize previously distinct parental lineages, including unique geographic or ecological variants (Todesco et al., 2016; Goulet et al., 2017). Hybridization affects the integrity of species boundaries and promotes phylogenetic discordance, confusing our understanding of the observed diversification patterns (Mallet et al., 2016; Stubbs et al., 2023). As a result, the reticulate processes challenge the way how we perceive species richness and classify genetic variation, the fundamental concepts for evolutionary biology and biodiversity conservation.

Genetic footprints could nowadays shed more light on the role that hybridization plays in shaping nature’s diversity (Moran et al., 2021; Porretta and Canestrelli, 2023). The evolutionary consequences of gene flow could be particularly pronounced in regions that experienced profound paleoecological changes and are now important biodiversity hotspots (Flantua et al., 2019; Schley et al., 2022; Wu et al., 2022a). Past climate fluctuations triggered substantial ecological disturbances in the high-elevation zone, promoting repeated elevation shifts and flickering connectivity of habitats occupied by alpine specialists (Flantua et al., 2019; Carruthers et al., 2024; Kandziora et al., 2024). Although the influence of temperature changes on mountain biota is well established, different sets of factors could drive species richness and shape fine-scale diversity patterns at the regional scale (Elsen and Tingley, 2015; Testolin et al., 2021). Moreover, multiple isolation periods intertwined with periods of species range overlaps could favor various genetic responses among alpine organisms, resulting in a wide array of evolutionary outcomes, among them hybridization after secondary contact (Antonelli et al., 2018; Rahbek et al., 2019; Schley et al., 2022). Since India-Eurasia collision, uplift and further dramatic geo-climatic changes promoted unprecedented plant radiations over one of the currently largest orogens in the world (Mosbrugger et al., 2018; Muellner-Riehl, 2019; Ding et al., 2020). The “Roof of the World”, as the highest parts of High Mountain Asia are commonly called, is therefore one of the most extraordinary areas to determine how hybridization affected diversification and population dynamics of high-mountain organisms (Wu et al., 2022a; Sinaga et al., 2024).

Gene flow events and complex patterns of reticulate evolution are common phenomena among flowering plants, blurring species boundaries, and confusing their identification (Soltis and Soltis, 2009; Mallet et al., 2016). One of the most complicated processes of microevolution and speciation is observed in the family of grasses (Poaceae), which demonstrate a wide variety of ploidy levels and ubiquity of hybridization events (Tkach et al., 2020; Gallaher et al., 2022). In particular, species-rich grass genera pose a particular challenge for taxonomy and phylogenetics, as observed, for example, in alkali grasses, *Puccinellia* Parl. (Tzvelev, 1976; Davis and Consaul, 2007; Consaul et al., 2010). This widely distributed genus shows tolerance to alkaline and/or saline conditions and is characterized by a large taxonomic and ecological diversity, which encompasses a wide range of latitudinal and elevation gradients (Soreng et al., 2022). *Puccinellia* is already regarded as a model system for deciphering the mechanisms responsible for salinity tolerance in halophilic grasses (Guo et al., 2020, Guo et al., 2021). Moreover, successful colonization of saline habitats throughout the Northern Hemisphere coupled with extensive species radiation since the Pliocene make *Puccinellia* a promising system to broaden our understanding of the microevolutionary processes under the changing climate and their role in the formation and/or extinction of plant species.

Alkali grasses are a core component of saline wetlands, moist grasslands, and turfs in the alpine biome of Central Asia (Liang et al., 2006; Świerszcz et al., 2021; Wróbel et al., 2023). This unique group of *Puccinellia* comprises halophilic and high-elevation extremophytes that occupy saline habitats between 2,000 and 5,500 m a.s.l., primarily in the Mountains of Central Asia (including the Mountains of Central Asia biodiversity hotspot – the Tian Shan and the Pamir Mountains), western Himalayas and western Qinghai-Tibetan Plateau (Ovchinnikov and Chukavina, 1957; Dickoré, 1995; Liang et al., 2006; Cope, 2011). Although the alpine species of *Puccinellia* are one of the most emblematic extremophytes of High Mountain Asia, their evolutionary history has only recently begun to attract more attention (Wróbel et al., 2023). Despite problematic identification and unknown phylogeny, to date, no large-scale study has examined the relationships among alpine *Puccinellia* and permeability of species boundaries. Hybridization may be an essential driver of speciation among high mountain *Puccinellia* given the frequent gene flow events detected among their lowland relatives (Steen et al., 2004; Davis and Consaul, 2007; Consaul et al., 2010; Gnutikov et al., 2020; Soreng et al., 2022; Kúr et al., 2023). In this study, we combine morphology and genomic data to investigate evolutionary patterns among alpine alkali grasses in the Pamir Mountains, the diversity center of the genus in the Mountains of Central Asia (Ovchinnikov and Chukavina, 1957; Dickoré, 1995; Liang et al., 2006; Lazkov and Sultanova, 2014).

Inconsistent approaches to the classification of morphological species have led to many taxonomic confusions and misidentifications among botanists who have so far aimed to investigate alpine *Puccinellia*. As a consequence, it is even challenging to determine how many species of alkali grasses currently occur in the Pamir Mountains (Ovchinnikov and Chukavina, 1957; Ikonnikov, 1963; Dickoré, 1995; Nowak et al., 2020). Therefore, we focused primarily on the core Pamir species that we were able to collect during field research and identify as matching to the descriptions of the typical morphotypes across different sources, particularly: *P. hackeliana* (V.I.Krecz.) V.I.Krecz. ex Drobow, *P. humilis* (Litv. ex V.I.Krecz.) Bor, *P. pamirica* (Roshev.) V.I.Krecz. ex Ovcz. & Czukav., *P. pauciramea* (Hack.) V.I.Krecz. ex Ovcz. & Czukav. and *P. vachanica* Ovcz. & Czukav. We also included verified herbarium specimens of *P. himalaica* Tzvelev and *P. subspicata* V.I.Krecz. ex Ovcz. & Czukav., which were collected in the Pamir Mountains in the 20th century. To provide a broader background for our inference, we added the specimens of *P. tianschanica* (Tzvelev) Ikonn., an alpine species described from the adjacent Tian Shan (Tzvelev, 1976). We included lowland specimens of *P. tenuiflora* (Griseb.) Scribn. & Merr. and *P. schischkinii* Tzvelev, two species that were earlier claimed to occur in the Pamir Mountains (Ikonnikov, 1963). Moreover, we incorporated several taxa collected in the Central Asian mountains, which may be associated with *P. distans* agg.: *P. gigantea* (Grossh.) Grossh., *P. glauca* (Regel) V.I.Krecz. and *P. hauptiana* (Trin. ex V.I.Krecz.) Kitagawa (Krechetovich, 1934). Lastly, we added one sample of *P. distans* (Jacq.) Parl. from Central Europe for comparative purposes. Taxonomic diversity and evolutionary complexity in a widely distributed *P. distans* agg. are a separate research challenge (Davis and Consaul, 2007; Kúr et al., 2023), remaining beyond the main focus of this study. Here, we put special emphasis on unraveling the origins of *P. vachanica*, a taxon with a poorly recognized distribution range and unclear status, which was described from the lower Wakhan Corridor in the southern edges of the Pamir Mountains (Ovchinnikov and Chukavina, 1957). *Puccinellia vachanica* has been inconsistently classified among different sources, in which some authors raised it to the rank of species, while others assigned it to *P. pamirica* as a synonym or subspecies under the name *P. pamirica* subsp. *vachanica* (Ovcz. & Czukav.) Tzvelev (Ovchinnikov and Chukavina, 1957; Tzvelev, 1976; Dickoré, 1995; Liang et al., 2006; WFO, 2024). During our field research, we observed individuals of *P. vachanica* in the lower part of the Wakhan Corridor (in Tajikistan), where it was reported earlier by Ovchinnikov and Chukavina (1957), as well as beyond the previously known distribution range of the species across the Pamir Mountains, in multiple high-elevated localities throughout the Pamir Plateau (in Tajikistan). The discovered populations of *P. vachanica* largely overlapped with the localities occupied by other species of *Puccinellia*, particularly *P. pamirica*. While selecting typical specimens of *P. pamirica* for population genetic analyses in our previous work (Wróbel et al., 2023), we found that *P. vachanica* is closely related to *P. pamirica* and could have originated as its interspecific hybrid.

Here, we addressed the following questions: (1) What are the phylogenetic relationships among alpine species of *Puccinellia* that occur in the Pamir Mountains? (2) Has interspecific hybridization played a role in the formation and/or extinction of particular species? (3) What are the origins of *P. vachanica*? Our goal was also to evaluate past spatio-temporal dynamics, which could have shaped development of the currently observed genetic and distribution patterns among alpine alkali grasses. Considering our primary focus on the Pamir Mountains, a thorough taxonomic and cytogeographic investigation of alpine alkali grasses is beyond the scope of this study. However, our genomic insight supported by morphological examination may serve as a reference point for a comprehensive future inventory of alpine *Puccinellia* throughout High Mountain Asia. Our study broadens understanding on how interspecific hybridization affects genetic diversity and species richness of alkali grasses in the alpine biome and contributes to informing conservation management in a Central Asian biodiversity hotspot.

## 2 Materials and methods

### 2.1 Study group

In this study, we examined all taxa previously reported from the Pamir Mountains and their adjacent regions (Ovchinnikov and Chukavina, 1957; Ikonnikov, 1963; Dickoré, 1995), which we were able to collect during our field research and verify while doing revision of the herbarium materials, including type specimens (LE, M, MSB, KRA, KRAM, TAD, FRU; acronyms after Index Herbariorum at https://sweetgum.nybg.org/science/ih/): *P. hackeliana*, *P. himalaica*, *P. humilis*, *P. pamirica*, *P. pauciramea*, *P. schischkinii*, *P. subspicata*, *P. tianschanica*, *P. tenuiflora*, *P. vachanica*, and *P. distans* agg. which we treated as a widespread species-complex for the purpose of this study, including morphotypes related to *P. distans*, *P. gigantea*, *P. glauca*, and *P. hauptiana*. The studied group is a mixed-ploidy system, in which alkali grasses are represented primarily by diploids and tetraploids (Sokolovskaya and Probatova, 1975; Tzvelev, 1976; Guinochet and Lefranc, 1981; Zakharyeva, 1985; Yan et al., 1989; Zakharjeva, 1993; Probatova et al., 2013; Kumar et al., 2018). However, higher ploidies were also reported for *P. hackeliana* and *P. distans* agg (Hughes, 1976; Tzvelev, 1976; Hughes and Halliday, 1980; Davis and Consaul, 2007; Kúr et al., 2023). The full list of ploidy levels reported for each taxon is shown in **Supplementary Table 1**. The taxonomic nomenclature was given after *Flora of China* (Liang et al., 2006).

During our field research in Central Asia between 2016 and 2023, we collected extensive plant material of all *Puccinellia* species that occurred in the multiple visited localities. We mainly explored the Central Tian Shan, the Pamir Plateau along its latitudinal axis, and the upper Panj River valley from the confluence of the Pamir and Wakhan rivers downslope (the lower part of the Wakhan Corridor). The reference specimens examined in this study were deposited in the KRA herbarium of the Jagiellonian University, Kraków, Poland (**Supplementary Table 2**).

### 2.2 SNP discovery and genotyping

We extracted genomic DNA from dried leaf tissues using the Genomic Mini AX Plant Kit (A&A Biotechnology, Poland), followed by quality control on agarose gel electrophoresis as well as quantity measure and purity check on the NanoDrop One (Thermo Scientific, USA). Samples with a DNA concentration of 95 ng/µL were sent to the Diversity Arrays Technology (DArT) Pty Ltd (Canberra, Australia) for sequencing and marker identification. Co-dominant single nucleotide polymorphism (SNP) markers were generated by the DArTseq™ platform, which uses complexity reduction methods, fragment size selection, and high-throughput sequencing optimized for a target species. Unlike other SNP-typing methods, DArT performs well in species with varying levels of ploidy. This effectiveness can be attributed, at least in part, to the use of high-fidelity restriction enzymes for SNP detection in DArT, rather than the dependence on primer annealing (Kilian et al., 2012). In this study, potential polyploid genotypes were subsampled into the biallelic format to meet the assumptions of the downstream analyses. A description of the discovery of SNP markers in *Puccinellia* is available in **Supplementary Presentation 1**.

### 2.3 Phylogenetic analyses

#### 2.3.1 DArTseq-based phylogeny

The SNP dataset was handled in the R environment (R Core Team, 2022; RStudio Team, 2022) using the dartR R-package with required dependencies (Mijangos et al., 2022). We selected one individual of *P. pamirica* per locality from our full dataset to maintain a more balanced sampling among the examined species (**Supplementary Table 2**). In total, 100 individuals were used for phylogenetic reconstruction.

First, we inferred a phylogenetic tree of the examined *Puccinellia* species using Bayesian Markov chain Monte Carlo analysis in MrBayes ver. 3.2.6 (Ronquist et al., 2012). The filtering procedure of the DArTseq dataset for the phylogenetic analyses included the following steps: 1) maintaining loci with reproducibility of 100%, 2) maintaining loci with a call rate of 100% to remove all missing data, 3) maintaining individuals with a call rate of 100% (all individuals preserved), 4) removing monomorphic loci, 5) filtering out secondary SNPs in order to use only unlinked SNPs – one randomly selected SNP was maintained per each locus that had more than one SNP detected. Stringent filtering criteria were used to account for the computational time required by Bayesian analysis. A final dataset consisted of 1,695 loci successfully genotyped among 100 examined individuals. We reconstructed an unrooted tree of the examined *Puccinellia* species since no outgroup taxon was analyzed via the DArTseq in this study. The best-fit substitution model was selected using MrModeltest ver. 2.3 (Nylander, 2004) and PAUP* ver. 4.0a169 (Swofford, 2002), which determined the SYM+G model as the best choice according to the AIC criterion. Two independent runs of four chained searches were performed for 20,000,000 generations, sampling every 500 generations, which was sufficient to reach the average standard deviation of the split frequencies below 0.01 (0.006). A 25% burn‐in was applied before constructing a 70% majority rule consensus tree and calculating Bayesian posterior probabilities. Clades with lower support values were presented as unresolved. The phylogenetic network was visualized in iTOL ver. 6 (Letunic and Bork, 2021) followed by further customization in Inkscape ver. 1.3.2 www.inkscape.org

Second, we inferred a phylogenetic tree of the DArTseq SNP panel using maximum-likelihood (ML) in IQ-TREE ver. 1.6.12 (Nguyen et al., 2015). The filtering procedure of the DArTseq dataset was modified in comparison to Bayesian inference by applying less stringent criteria for a locus call rate, which was performed to evaluate the potential impact of the applied filtering threshold on the inferred tree topology. We maintained loci with a call rate of 95% during Step 2), which allowed us to retain 7,548 loci in the final dataset. We used ModelFinder to select the best-fit substitution model to the data (Kalyaanamoorthy et al., 2017) and the TIM3+F+R2 model was selected according to the BIC criterion. To evaluate the support for branches of the inferred phylogeny, we used the Shimodaira–Hasegawa-like approximate likelihood ratio test (SH-aLRT) and the Ultrafast Bootstrap Approximation (UFBoot) (Guindon et al., 2010; Hoang et al., 2018). The UFBoot converged after 347 iterations, and the estimate of Bootstrap correlation coefficient of split occurrence frequencies was 0.996. A particular clade was preserved in the tree topology when it had the SH-aLRT value reaching at least 80% and the UFBoot value of at least 95%.

#### 2.3.2 Chloroplast phylogeny

We examined selected markers of chloroplast DNA (cpDNA) to determine phylogenetic relationships and the direction of potential hybridization between the examined species. We chose two non-coding chloroplast regions that appeared useful in the previous phylogenetic studies focused on the genus *Puccinellia* (Consaul et al., 2010): the *rpl16* intron and the *rpoB-trnC* intergenic spacer.

To amplify the *rpl16* intron, we used primers F71 (5’-GCT ATG CTT AGT GTG TGA CTC GTT G-3’) and R1661 (5’-CGT ACC CAT ATT TTT CCA CCA CGA C-3’) (Small et al., 1998). For the *rpoB-trnC* intergenic spacer, we used primers ANU_cp036-R RtrnC (GCA) (5’-TGC AGT CCC CTG CCT TAC-3’) and ANU_cp035-L FrpoB (Ebert and Peakall, 2009), which was customized in this study to better align with *Puccinellia* species by changing the original 16th base from A to G (5’-TGT GGA CAT TCC CTC GTT TC-3’). Adjustment was made based on reference *rpoB* gene sequences available in two complete chloroplast genomes of *P. distans* and *P. nuttalliana* (GenBank: MW044608.1 and KM974750.1).

Both chloroplast markers were amplified in a total volume of 25 µl, containing: 2.5 μl of 10× DreamTaq Green Buffer (including 20 mM MgCl_2_; Thermo Scientific), 0.5 μl dNTPs (10 mM), 0.5 μl of each primer (10 pmol/μl), 2 μg of bovine serum albumine (BSA), 2.5 U of DreamTaq DNA Polymerase (Thermo Scientific), 1 μl of genomic DNA isolate (working concentration of 10 ng/μl, diluted in water) and ultrapure water added up to 25 µl. We used a stock solution of genomic DNA in case amplification failed at a diluted working concentration.

To optimize PCR conditions, we first tested the effectiveness of primer annealing at different temperature levels in a range of 50 to 58°C to determine the most effective conditions. For the final analyses, the PCR mixtures were initially heated at 94°C for 10 min before 35 cycles of PCR amplification performed on the Mastercycler DNA thermal cycler (Eppendorf, Germany); one PCR cycle consisted of denaturation at 94°C for 45 s, primer annealing at 58°C (*rpl16* intron) or 51°C (*rpoB-trnC* intergenic spacer) for 1 min, and extension at 72°C for 2 min; the reactions were completed with the final elongation step at 72°C for 7 min. Reactions without DNA were used as negative controls. The PCR products were sent to an external company (Genomed, Poland) for purification and paired-end Sanger sequencing. For the *rpl16* intron, only primer R1661 enabled sequence acquisition, while for the *rpoB-trnC* intergenic spacer both primers provided good quality sequence reads. Chromatograms were manually verified, and sequences were aligned using BioEdit ver. 7.2.5 (Hall, 1999). We obtained reliable reads of the partial *rpl16* intron sequences (807 bp long) and the partial *rpoB-trnC* intergenic spacer sequences (926 bp long) for 72 individuals of the studied *Puccinellia* species (**Supplementary Table 2**).

We inferred the phylogenetic tree of the examined cpDNA markers in *Puccinellia* species using Bayesian Markov chain Monte Carlo analysis in MrBayes ver. 3.2.6 (Ronquist et al., 2012). We treated the *rpl16* intron and the *rpoB-trnC* intergenic spacer as one partition since both markers are non-coding chloroplast regions. The tree was rooted by the plastid sequences of *Sclerochloa dura* (extracted from the complete chloroplast genome; GenBank: MT094329.1), which is considered the genus most closely related to *Puccinellia* (Soreng et al., 2022). The best-fit substitution model was selected using MrModeltest ver. 2.3 (Nylander, 2004) and PAUP* ver. 4.0a169 (Swofford, 2002), which determined the SYM+G model as the best choice according to the AIC criterion. Two independent runs of four chained searches were performed for 500,000 generations, sampling every 500 generations, which was sufficient to reach the average standard deviation of the split frequencies below 0.01 (0.009). A 25% burn‐in was applied before constructing a 50% majority rule consensus tree and calculating Bayesian posterior probabilities. The full topology of the inferred Bayesian tree was visualized in iTOL followed by further customization in Inkscape. Additionally, we performed phylogenetic inference using maximum-likelihood in PhyML 3.0 (Guindon et al., 2010). We used Smart Model Selection to select the best-fit substitution model to the data (Lefort et al., 2017). The HKY85 model was selected for the cpDNA markers. We applied SH-aLRT to evaluate the support for branches of the inferred phylogeny (Guindon et al., 2010). We also reconstructed the haplotype network of the SNPs detected in the *rpl16* intron and the *rpoB-trnC* intergenic spacer using the TCS approach (Clement et al., 2002) in POPART ver. 1.7 (Leigh and Bryant, 2015).

### 2.4 Hybrid detection

We used a wide array of programs and different subsets of individuals to evaluate genetic clusters and potential admixture signals among the examined alkali grasses. First, we added more individuals of *P. pamirica* to increase the potential resolution of the analyses devoted to detect more fine-scale evolutionary processes compared to the phylogenetic inference (**Supplementary Table 2**). In total, the full dataset included 160 individuals. We used fastSTRUCTURE (Raj et al., 2014) as the first clustering algorithm to determine genetic groups in the dataset. We tested a range of group numbers (K) from 1 to 10 in 10 replicate runs using a simple prior. In fastSTRUCTURE, the optimal K is considered to fall between model complexity that maximizes marginal likelihood and model components used to explain structure in data. The bar chart of inferred group assignment among individuals was prepared using the ggplot2 R-package (Wickham, 2016). Moreover, we used TreeMix ver. 1.13 (Pickrell and Pritchard, 2012) to reveal potential gene flow among the examined *Puccinellia* species. TreeMix reconstructs the maximum likelihood tree of the evolutionary relationship and recovers potential admixture events between *a priori* defined groups. We performed TreeMix modeling using seven homogeneous clusters distinguished by fastSTRUCTURE. We set a window size (k) for 1 and 50 SNPs and performed 10 repetitions per each value of migration events (m) ranging from 1 to 10. We evaluated the optimal number of migrations in OptM ver. 0.1.6 (Fitak, 2021). We recognized a migration event as reliable when it was recovered in the optimal models for both window sizes. The filtering procedure for fastSTRUCTURE and TreeMix analyses based on the full dataset (160 individuals) included the following steps: 1) maintaining loci with reproducibility of 100%, 2) maintaining loci of a call rate of 100% to remove all missing data, 3) maintaining individuals of a call rate of 100% (all individuals preserved), 4) removing monomorphic loci, 5) filtering out secondary SNPs in order to use only unlinked SNPs. The complete dataset was used to account for the particular sensitivity of the TreeMix analysis to missing data (Pickrell and Pritchard, 2012) and to maintain the possibility of a direct comparison between the obtained TreeMix output and results of cluster analysis. A final dataset consisted of 1,528 loci successfully genotyped among 160 examined individuals.

Second, we performed a fastSTRUCTURE analysis on the dataset without the only available sample of *P. himalaica*. The goal of such a procedure was to evaluate the effects of sample exclusion on cluster inference. The input dataset included 159 individuals and retained 1,630 loci after the same filtering strategy as used previously. We tested a range of group numbers (K) from 1 to 10 in 2 replicate runs using simple prior. Third, we used STRUCTURE ver. 2.3.4 (Pritchard et al., 2000) to infer population structure in the examined alkali grasses. Here, we used only one sample per each major clade reconstructed in the phylogenetic inferences. The goal of this procedure was to evaluate the outcome of genetic clustering when only a single sample is used for a particular taxon. The input dataset consisted of 11 individuals and retained 3,051 loci after the same filtering strategy as used previously. STRUCTURE was run using the admixture model with correlated allele frequencies. For each of the values of K from 1 to 11, 10 runs were performed. Each run consisted of 100,000 burn-in MCMC iterations followed by 100,000 MCMC post burn-in iterations. The other settings remained default. The results were analyzed using the POPHELPER 2.3.1 R-package (Francis, 2017) to determine the most optimal K number taking into account a peak of Evanno’s ΔK value (Evanno et al., 2005).

### 2.5 Morphology

Morphological examination was performed to support the interpretation of the genetic data. We analyzed selected morphological characters of the studied *Puccinellia* species to compare their phenotypic similarity with the resolved genomic relationships. We focused particularly on characters related to the morphology of panicles and florets, which appeared informative in the previous taxonomic research on alpine alkali grasses (Ovchinnikov and Chukavina, 1957; Liang et al., 2006). Lowland individuals of *P. schischkinii* were excluded from a detailed examination due to their distinct morphology and a more distant genetic relationship with *P. vachanica*. We used principal coordinates analysis (PCoA) to examine the morphological patterns among the studied alkali grasses. The correlation between variables was checked using Pearson’s coefficient in the stats R-package (R Core Team, 2022). Variables that strongly correlated with another variable, i.e. having coefficients exceeding 0.7, were removed. The seven remaining characters included both quantitative and qualitative features. Therefore, Gower’s similarity index was used to construct a distance matrix as implemented in the proxy R-package ver. 0.4-27 (Meyer and Buchta, 2022). The PCoA was performed on the distance matrix using the ape R-package ver. 5.7-1 (Paradis et al., 2004). The complete morphological dataset is available as **Supplementary Table 3**.

### 2.6 Spatial genetic structure

We used DArTseq SNP markers to analyze the genetic variation patterns among the specimens assigned to *P. vachanica*, which were discovered in numerous localities throughout the Pamir Mountains. As we determined in our previous work (Wróbel et al., 2023), *P. pamirica* has developed detectable patterns of genetic differentiation across the Pamir Mountains. Therefore, the objective of this analysis was to examine whether *P. vachanica* also demonstrates differentiation signals similar to *P. pamirica* in the research area.

The SNP dataset consisted of 117 individuals, 81 of *P. pamirica* and 36 of *P. vachanica*, which were all isolated from fresh material collected during our field research and did not show substantial levels of missing data as old herbarium materials. Therefore, we applied less stringent filtering criteria for a locus call rate to preserve more loci and provide better discriminatory power for population inference. The dataset was filtered using the dartR R-package (Mijangos et al., 2022), including the following steps: 1) maintaining loci with reproducibility of 100%, 2) maintaining loci with a call rate of 75%, 3) maintaining all individuals (the minimum value of a call rate by individual was 0.895), 4) removing monomorphic loci, and 5) filtering out secondary SNPs in order to use only unlinked SNPs. A final dataset consisted of 11,519 loci.

We performed the phylogenetic analyses using ML in IQ-TREE ver. 1.6.12 (Nguyen et al., 2015) to examine the potential genetic structure of *P. vachanica* compared to *P. pamirica*. We used ModelFinder to select the best-fit substitution model to the data (Kalyaanamoorthy et al., 2017) and the TPM3u+F+R9 model was selected according to the BIC criterion. To evaluate the support for branches of the inferred phylogeny, we used the SH-aLRT test and UFBoot (Guindon et al., 2010; Hoang et al., 2018). The UFBoot converged after 185 iterations and the estimate of Bootstrap correlation coefficient of split occurrence frequencies was 0.995. A particular clade was preserved in the tree topology when it had the SH-aLRT value reaching at least 80% or the UFBoot value of at least 95% to maintain satisfactory resolution.

### 2.7 Lineage diversification and hybrid origin scenarios

We used approximate Bayesian computation (ABC) with supervised machine learning in coalescence-based DIYABC Random Forest ver. 1.2.1 (Collin et al., 2021) to test six alternative hypotheses on the history of lineage diversification and hybrid origins of *P. vachanica*. As the key time horizon for the analyzed events, we assumed the local Last Glacial Maximum (local LGM) in the Pamir Mountains estimated between ~50,000–100,000 years ago (Owen and Dortch, 2014), the potentially important driver for profound ecological shifts in this Central Asian region during the Late Pleistocene (Wróbel et al., 2023). For time interpretation, we considered a generation time of 2 years. As “post-local LGM” events we assumed those that occurred after the local LGM, between 10 and 25,000 generations (t_1_ and t_2_ parameters; uniform prior distribution range; t_1_<t_2_ when used together in a scenario), while as “local LGM” events we considered those that occurred during the local LGM, between 25,000 and 50,000 generations (t_3_ and t_4_ parameters; uniform prior distribution range; t_3_<t_4_ when used together in a scenario). The split of the ancestral lineages of the parental species remains unknown and was assumed to occur between 100,000 and 200,000 generations (t_5_ parameter; uniform prior distribution range). The crown node of *Puccinellia* comprising all extant alkali grasses has been estimated between 0.64–2.29 My (Hoffmann et al., 2013) or 2.39–4.12 My (Soreng et al., 2022), depending on the approach used and the inferred placement of *Sclerochloa* in the analyzed datasets. Therefore, we also tested 750,000 and 1,250,000 generations as the upper value of the prior range for t_5_ to account for the potentially earlier split of the ancestral lineages of the parental species. The same scenario was consistently determined as the best supported and the main conclusions derived from the DIYABC-RF analysis remained intact regardless of the priors used for t_5_. The admixture rate derived from *P. pamirica* during interspecific hybridization with the second parental species revealed in genetic structure analyses and TreeMix was set between 0.01 and 0.99 (r_1_ parameter).

Our primary goal was to determine the number of past gene flow events between the parental species (single-event vs multiple-event origin hypotheses) that led to the formation of *P. vachanica* and the emergence of its spatial genetic patterns. Three scenarios (1–3) aimed to examine potential multiple-event origins of hybrids and different timing of lineage diversification and gene flow. Scenario 1 assumed the post-local LGM intraspecific diversification of *P. pamirica* (t_2_) followed by its multiple post-local LGM interspecific hybridization events (t_1_). Scenario 2 assumed intraspecific diversification of *P. pamirica* during the local LGM (t_4_) followed by its multiple interspecific hybridization events during the local LGM (t_3_). Scenario 3 assumed intraspecific diversification of *P. pamirica* during the local LGM (t_4_) followed by its multiple post-local LGM interspecific hybridization events (t_2_). Three other scenarios (4–6) were designed to examine a potential single-event formation of a hybrid lineage, which then evolved independently from its parental species. Scenario 4 assumed a post-local LGM hybridization event (t_2_) followed by independent post-local LGM diversification of hybrids and their parental species (t_1_). Scenario 5 assumed one hybridization event during the local LGM (t_4_) followed by independent diversification of hybrids and their parental species during the local LGM (t_3_). Scenario 6 assumed one hybridization event during the local LGM (t_4_) followed by independent post-local LGM diversification of hybrids and their parental species (t_2_).

To represent the fine-scale genetic structure of *P. pamirica* and *P. vachanica* in the model, we used two approaches. First, we focused on the division of *P. pamirica* and its hybrids into two main geographic groups established in the Pamir Mountains, northern and southern, separated in the center of the Pamir Plateau (Wróbel et al., 2023). This model was based on 118 individuals and is called a ‘North/South cluster model’ along the article. Second, we analyzed a model in which only one region was selected per each geographic cluster where both *P. pamirica* and its hybrids occurred. We chose Karakul lake for the northern cluster (populations 29–31 in **Supplementary Table 2**) and the middle section of the Pamir River for the southern cluster (populations 10 and 11 in **Supplementary Table 2**). This model was based on 27 individuals and is called a ‘North/South population model’ throughout the article. For both subsets of individuals, the SNP dataset was filtered using the dartR R-package, including the steps previously specified in Section 2.4 ‘Hybrid Detection’. Then, we filtered out loci that were monomorphic among all *P. pamirica* and among all hybrids to preserve only the most polymorphic loci in both datasets. We set the minimum allele frequency to 5% (MAF=0.05). The dataset retained 338 SNPs in the North/South cluster model and 181 in the North/South population model. We generated from 2,000 to 20,000 simulations per scenario, which is a recommended range for a model choice in DIYABC-RF (Collin et al., 2021). The same scenario was consistently determined as the best supported regardless of the number of simulations used. For the final model choice and parameter estimates, we used 10,000 simulations per scenario. We performed 10 replicate RF-analyses with 500 RF-trees based on 10 different reference tables to establish the proportion of RF classification votes for each scenario and their mean posterior probability as a confidence measure of the scenario choice (Chapuis et al., 2020). Parameter estimates were derived from the best supported scenarios in both models. We estimated the median value and the 5% and 95% quantiles of parameter posterior distributions based on 10 replicate RF-analyses.

## 3 Results

### 3.1 DArTseq-based phylogeny

**Figure 1:**
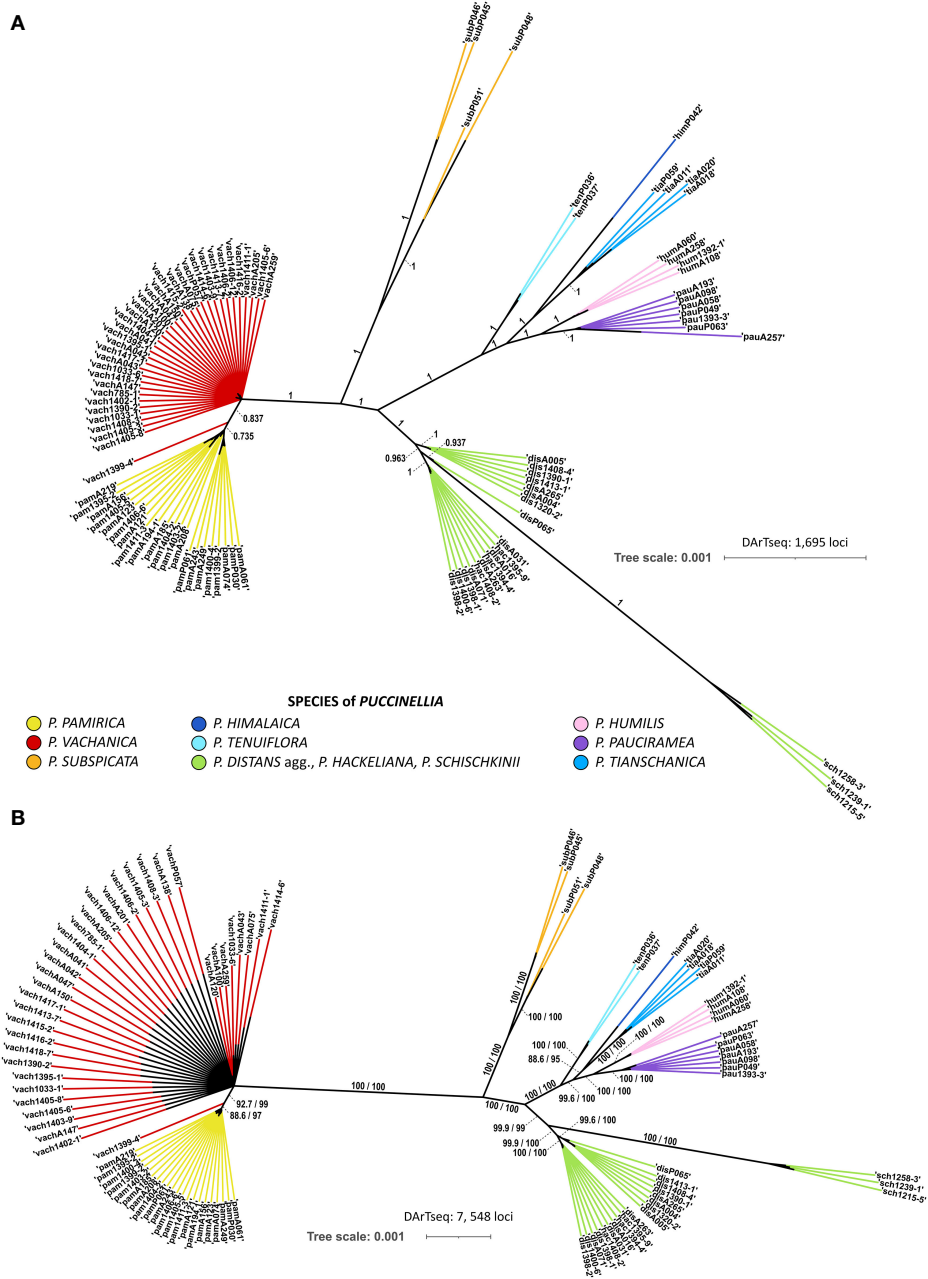
Genomic relationships among the examined *Puccinellia* species reconstructed from the single nucleotide polymorphism panel generated by the Diversity Arrays Technology sequencing (DArTseq) approach. **(A)** Phylogenetic unrooted tree reconstructed using Bayesian Markov chain Monte Carlo analysis in MrBayes ver. 3.2.6 based on 1,695 loci. The tree topology shows only clades which reached at least 0.7 of Bayesian posterior probability. Main evolutionary branches inferred by the Bayesian analysis were labeled with their support values. **(B)** Phylogenetic unrooted tree inferred using maximum-likelihood (ML) in IQ-TREE ver. 1.6.12 based on 7,548 loci. A particular clade was preserved in the tree topology when it has the Shimodaira–Hasegawa-like approximate likelihood ratio test (SH-aLRT) value reaching at least 80% and the Ultrafast Bootstrap Approximation (UFBoot) value of at least 95%. Main evolutionary branches inferred by the ML analysis were labeled with their support values (SH-aLRT/UFBoot).

Bayesian and ML phylogenetic reconstructions resolved congruent tree topologies and supported the occurrence of the same main evolutionary clades among the examined alkali grasses (**Figure 1**). The genomic SNP panel provided enough power to discriminate between most of the alpine species of *Puccinellia*. Both phylogenetic analyses distinguished four main evolutionary groups in the studied system of alkali grasses: 1) *P. pamirica* and *P. vachanica*, 2) *P. subspicata*, 3) *P. tenuiflora*, *P. humilis*, *P. himalaica*, *P. tianschanica*, and *P. pauciramea*, and 4) *P. distans* agg., *P. hackeliana*, and *P. schischkinii*. Except for *P. hackeliana* and *P. vachanica*, all other alpine species that occur in the Pamir Mountains were resolved as monophyletic clades and received strong support in both phylogenetic reconstructions, particularly *P. pamirica*, *P. subspicata*, *P. humilis*, *P. himalaica*, and *P. pauciramea*.

Phylogenetic analysis showed that the alpine alkali grasses of Central Asia did not form a major phylogenetic clade (**Figure 1**). The inferred tree topology revealed that some of the high-mountain alkali grasses shared clades with their lowland relatives. The position of *P. hackeliana* as a separate species was not supported by phylogenetic inference. The species was nested within one of two main clades comprising Central Asian individuals assigned to *P. distans* agg., suggesting its close relationship with this widely distributed species complex. Furthermore, the central European sample of *P. distans* (P065) was also resolved as closely related to Central Asian individuals attributed to *P. distans* agg. This indicates the maintenance of a strong phylogenetic signal despite the huge geographic distance observed currently among the individuals sampled from this evolutionary branch. The Siberian individuals of *P. tenuiflora* were clustered with four alpine species of alkali grasses that occur in the Mountains of Central Asia, namely *P. humilis*, *P. himalaica*, *P. tianschanica*, and *P. pauciramea*. Such placement uncovers a close phylogenetic relationship of these high-mountain species with their widely distributed relative, whose distribution range spans currently through Asia and covers mostly lowland and low-mountain regions.

Despite shared geographic affinity, the Pamir specimen of *P. himalaica* was not clustered with its close relatives collected in the same mountain range, namely the Pamir individuals of *P. humilis* and *P. pauciramea*. Rather, *P. himalaica* was reconstructed as a sister species to *P. tianschanica* sampled in the adjacent Tian Shan. Three other Central Asian alpine species, *P. subspicata*, *P. pamirica*, and *P. vachanica*, were resolved as strongly divergent from the other examined alkali grasses. Individuals assigned to *P. subspicata* showed a clear intraspecific division into two clades, which corresponded to their geographic location in the Pamir Mountains or Tian Shan. Although *P. vachanica* demonstrated a close relationship with *P. pamirica*, it maintained a clear distinction from its closest relative in phylogenetic inference. *Puccinellia vachanica* was neither nested within the clade of *P. pamirica* nor resolved as its sister species. According to the genomic SNP data, *P. vachanica* was resolved as a paraphyletic group in relation to *P. pamirica*.

### 3.2 Chloroplast phylogeny

**Figure 2:**
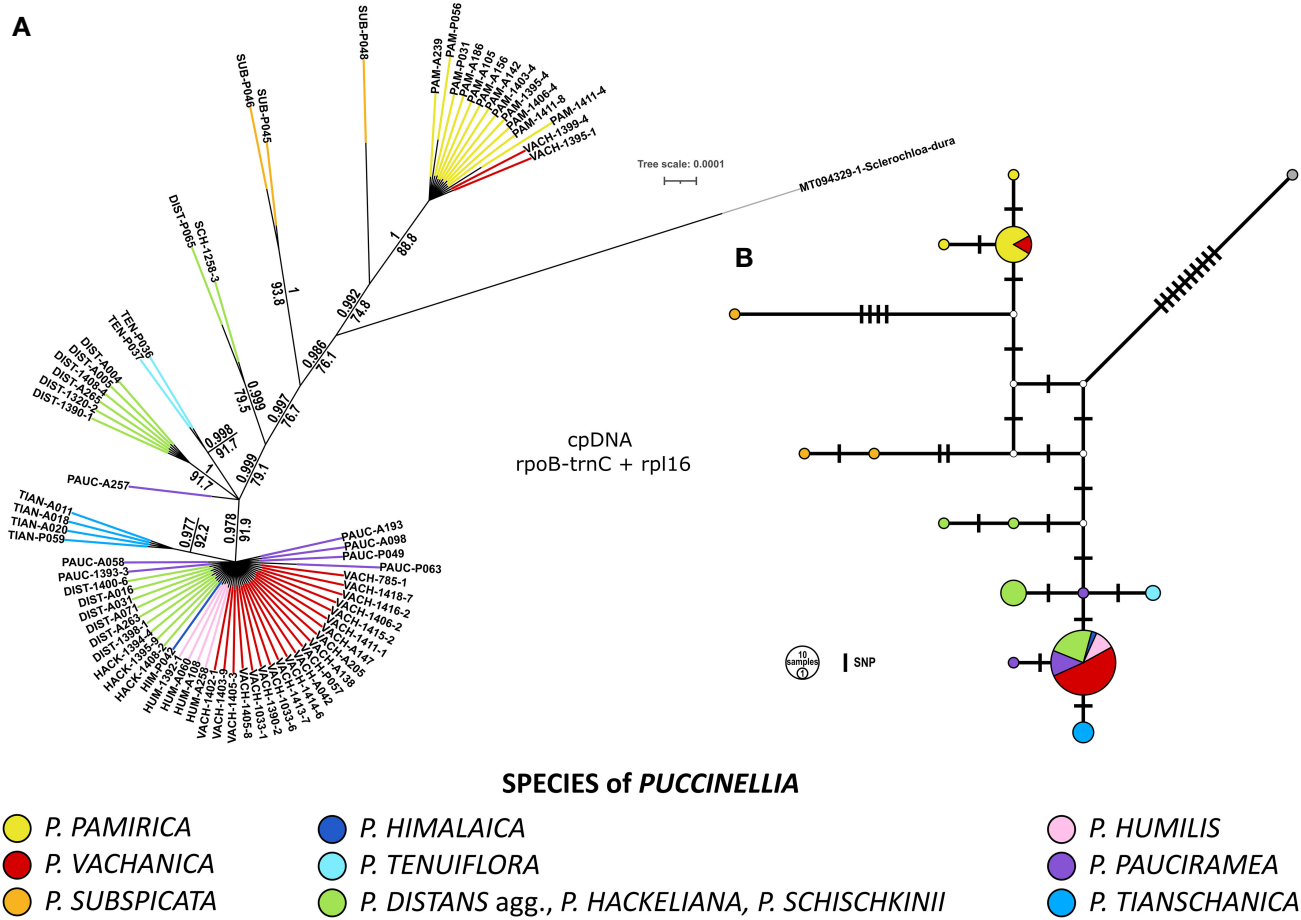
Genetic relationships among the examined *Puccinellia* species based on two non-coding chloroplast markers – *rpl16* intron and *rpoB-trnC* intergenic spacer. The sequences of *Sclerochloa dura* were used as an outgroup (GenBank: MT094329.1). **(A)** Phylogenetic tree inferred using Bayesian Markov chain Monte Carlo analyses performed in MrBayes ver. 3.2.6. Support values: Bayesian posterior probability (above a branch), Shimodaira–Hasegawa-like approximate likelihood ratio test performed for the inferred phylogeny using maximum-likelihood in PhyML 3.0 (below a branch). **(B)** Haplotype network of *rpl16* intron and *rpoB-trnC* intergenic spacer using the TCS approach in POPART ver. 1.7.

The combination of the *rpl16* intron and the *rpoB-trnC* intergenic spacer allowed us to differentiate between the main evolutionary lineages of alkali grasses previously reconstructed from the DArTseq genomic data (compare **Figures 1**, **2**). Bayesian inference and ML analysis supported the same topology of the phylogenetic tree among the studied *Puccinellia* species. Two plastid markers provided lower discriminatory power than DArTseq data and differentiated less than half of the examined alpine *Puccinellia* species from each other. However, the inclusion of an outgroup taxon, *Sclerochloa dura*, provided more phylogenetic context in the inferred tree topology, indicating the early divergence of *P. pamirica* and *P. subspicata* from the rest of the analyzed alkali grasses (**Figure 2**). In addition, one SNP discovered in the *rpl16* intron allowed us to distinguish *P. tianschanica* from all other high-mountain species of Central Asia. Apart from that SNP difference, two other mutations also supported this distinction, one 5bp insertion and one 6bp deletion unique for *P. tianschanica* among the individuals analyzed for *Puccinellia*. According to the plastid data, most alpine alkali grasses collected in the Mountains of Central Asia shared the same plastid sequences of the *rpl16* intron and the *rpoB-trnC* intergenic spacer, with the exclusion of six individuals related to *P. distans* agg. and two individuals of *P. pauciramea*. The chloroplast markers supported the division of Central Asian individuals assigned to *P. distans* agg. or *P. hackeliana* into two clades consistent with those reconstructed previously from the DArTseq data (compare **Figures 1**, **2**). *Puccinellia pauciramea* was not resolved as a monophyletic clade according to the plastid data. The species did not demonstrate cohesion of its chloroplast sequences, maintaining three different haplotypes in the species genetic pool that encompassed the Pamir Mountains (6 samples) and the Tian Shan (1 sample; P063). Moreover, we detected incongruent placement of *P. tenuiflora* and *P. vachanica* within the plastid and DArTseq-based phylogenies (compare **Figures 1**, **2**). Although *P. tenuiflora* was placed at different positions in the inferred tree topologies, it was consistently resolved as a monophyletic clade in both datasets. On the other hand, the plastid data revealed the polyphyly of the individuals assigned to *P. vachanica*. Most of these specimens shared the most common chloroplast haplotype detected among the alpine alkali grasses occurring in the Mountains of Central Asia. However, two individuals of *P. vachanica* (1395-1, 1399-4) were nested within the dominant haplotype of *P. pamirica*. Apart from the analyzed SNP differences, these plants also had one 6bp long insertion in the *rpoB-trnC* intergenic spacer, which was detected only in *P. pamirica* among the analyzed alkali grasses.

### 3.3 Hybrid detection

#### 3.3.1 DArTseq-based cluster analyses and gene flow reconstruction

**Figure 3:**
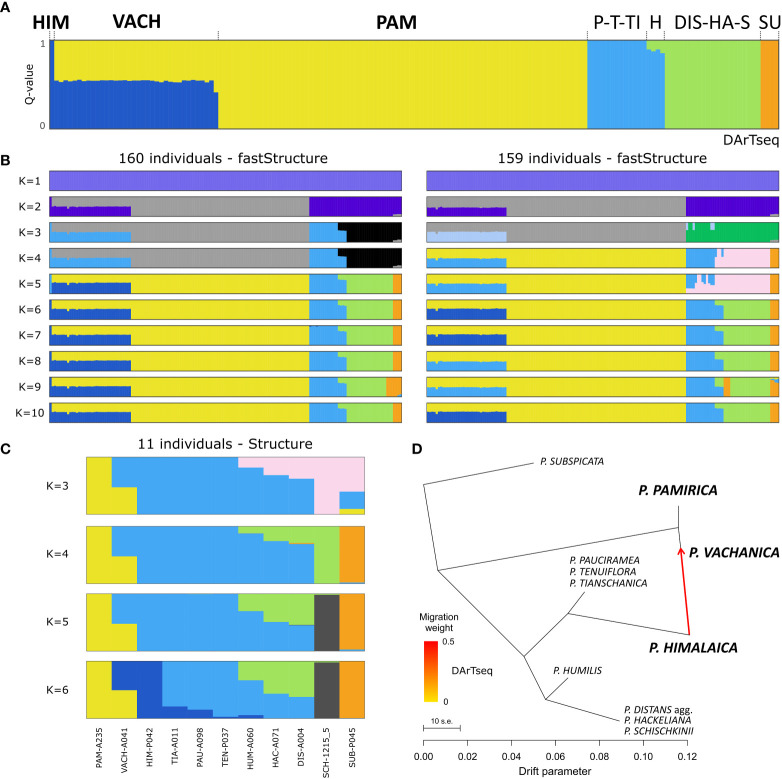
Cluster analyses and gene flow reconstruction among the examined *Puccinellia* species based on the genomic single nucleotide polymorphism panel generated by the Diversity Arrays Technology sequencing (DArTseq) approach. **(A)** Main evolutionary groups determined in fastSTRUCTURE using the full dataset (160 individuals). Species abbreviations: *P. himalaica* (HIM), *P. vachanica* (VACH), *P. pamirica* (PAM), clade of *P. pauciramea*, *P. tenuiflora*, and *P. tianschanica* (P-T-TI), *P. humilis* (H), *P. distans* agg., *P. hackeliana*, and *P. schischkinii* (DIS-HA-S), and *P. subspicata* (SU). **(B)** Main evolutionary groups determined in fastSTRUCTURE using the dataset with the exclusion of *P. himalaica* (159 individuals). **(C)** STRUCTURE results using one sample per 11 major clades reconstructed in the phylogenetic inference (see **Figure 1**). **(D)** Gene flow events recovered by TreeMix ver. 1.13 in the genetic network of the main evolutionary lineages among the examined *Puccinellia* species.

FastSTRUCTURE showed that the optimal number of groups to explain the genetic structure among the examined alkali grasses remains between four and 10 (**Supplementary Table 4**). However, none of the runs determined more than five proper groups when ghost clusters (with marginal contributions) were excluded from the results. The exploration of the results of the tested range of K values suggested the delimitation of genetic groups congruent with those inferred in the phylogenetic reconstructions: 1) *P. pamirica*, 2) *P. subspicata*, 3) *P. pauciramea*, *P. tenuiflora*, *P. tianschanica*, and *P. himalaica* (P-T-TI-HIM), 4) *P. distans* agg., *P. hackeliana*, and *P. schischkinii* (DIS-HA-S) (**Figures 3A, B**). In some cases*, P. himalaica* was distinguished as the additional group. FastSTRUCTURE reconstructed *P. humilis* as closely related to the P-T-TI-HIM group with potential small input from the DIS-HA-S group. All individuals of *P. vachanica* were assigned to *P. pamirica* with ~50% Q-value and in other half to the P-T-TI-HIM group or strictly to *P. himalaica*. When *P. himalaica* was excluded from the analysis (a dataset of 159 individuals), *P. vachanica* was still reconstructed as sharing assignment between two different groups (**Figure 3B**). One of these clusters was related to *P. pamirica*, while the other represented either the P-T-TI group, which comprises the most closely related species to *P. himalaica*, or a genetic group that was not inferred in any other individual in the analyzed reduced dataset. In STRUCTURE, the ΔK statistic indicated that three or five clusters could be the optimal values according to the local maxima of this metric (**Supplementary Figure 1**). Similarly to both fastSTRUCTURE analyses, *P. vachanica* was resolved in STRUCTURE as sharing assignment in *P. pamirica* and the P-T-TI-HIM group (**Figure 3C**). TreeMix determined potential gene flow signals between *P. himalaica* and *P. pamirica*, supporting the occurrence of their hybrids in the Pamir Mountains (**Figure 3D**). Such a genetic admixture was recovered in all TreeMix models irrespective of the settings used. According to TreeMix, the individuals classified as *P. vachanica* shared a similar contribution of genetic ancestry from *P. himalaica* and *P. pamirica* in the reconstructed species network.

#### 3.3.2 Direction of hybridization based on chloroplast DNA

We obtained reliable reads of the partial *rpl16* intron sequences and partial *rpoB-trnC* intergenic spacer sequences for 12 accessions of *P. pamirica*, 1 of *P. himalaica*, and their 22 hybrids (assigned to *P. vachanica*), detected earlier using the DArTseq SNP markers. The *rpl16* intron alone did not provide enough resolution to differentiate *P. pamirica* from *P. himalaica*. However, the *rpoB-trnC* intergenic spacer enabled distinguishing the haplotypes of these two species. The *rpoB-trnC* intergenic spacer differed between *P. pamirica* and *P. himalaica* by four SNPs, one 4 bp long insertion/deletion and one 6 bp long insertion/deletion. Phylogenetic and haplotype analyses revealed that these two species of *Puccinellia* hybridized in both directions (**Figure 2**). We determined that the crosses that received maternal genetic input from *P. himalaica* occurred frequently across the entire Pamir Plateau and in the lower Wakhan Corridor. On the other hand, hybrids with maternal ancestry assigned to *P. pamirica* were discovered only in two localities, one located in the southern and one in the northern part of the Pamir Plateau (1395-1, 1399-4).

### 3.4 Morphology

**Figure 4:**
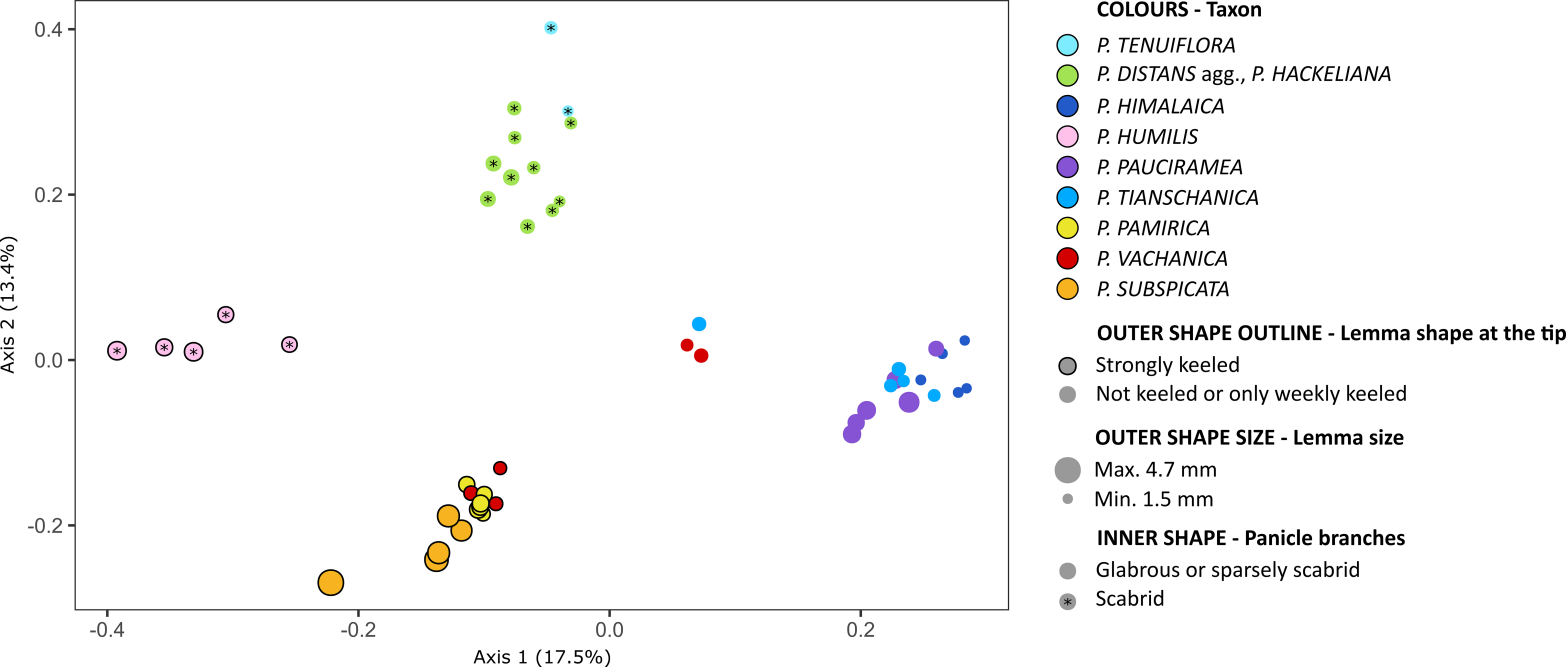
Morphological patterns detected among the studied *Puccinellia* species revealed by means of Principal Coordinates Analysis. The distance matrix was constructed using Gower’s similarity index based on seven morphological variables. Full morphological dataset is included in **Supplementary Table 3**. High-resolution photographs presenting panicle morphology of the alpine species are attached as **Supplementary Figures 2** and **3**.

The alpine species of *Puccinellia* could be distinguished from each other by a set of morphological characters (**Figure 4**; **Supplementary Figure 2**). Single characters are usually not sufficient to delimit a particular species due to detectable intraspecific variation in qualitative features and frequent overlaps of the value ranges for quantitative features measured among different species (**Supplementary Table 3**). Except *P. humilis*, the morphological patterns of the alpine species resembled their genomic relationships to a large extent (compare **Figures 1**, **4**). *Puccinellia pamirica* was grouped on the PCoA plot near *P. subspicata*, similarly to the DArTseq-based and chloroplast DNA inferences. These two species were primarily distinguished by their glabrous and strongly keeled lemmas, as well as narrow panicles with glabrous branches. Additionally, *P. subspicata* had by far the longest lemmas and anthers among all the alkali grasses studied. The PCoA analysis supported the morphological similarity of *P. pauciramea*, *P. tianschanica*, and *P. himalaica*, which were also resolved as closely related species in the DArTseq-based phylogeny and genetic structure analyses. These three species are characterized by spreading panicles with smooth branches and glabrous lemmas with rounded tips. *Puccinellia pauciramea* could be distinguished from *P. himalaica* and *P. tianschanica* by longer lemmas. From the latter two species, *P. himalaica* may be particularly identified by shorter lemmas and anthers compared to *P. tianschanica*. The species with scabrid panicle branches could be distinguished from each other by lemma morphology. *Puccinellia humilis* and most of the specimens attributed to *P. distans* agg. had hairy lemmas in contrast to glabrous *P. tenuiflora*. Moreover, the shape of lemma differentiated strongly keeled *P. humilis* from rounded *P. distans* agg. and *P. tenuiflora*. All individuals of *P. vachanica* had glabrous panicle branches and shorter lemmas than *P. pamirica*. *Puccinellia vachanica* was characterized by a high phenotypic diversity (**Supplementary Figure 3**) and was divided into two groups in the PCoA. Individuals with narrow panicles and keeled lemmas were placed near *P. pamirica*. On the other hand, the individuals with spreading panicles and more rounded lemmas showed more similarity to the group including *P. pauciramea*, *P. tianschanica*, and *P. himalaica*. Considering the length of lemma, all individuals of *P. vachanica* appeared intermediate between *P. pamirica* and *P. himalaica*.

### 3.5 Distribution

**Figure 5:**
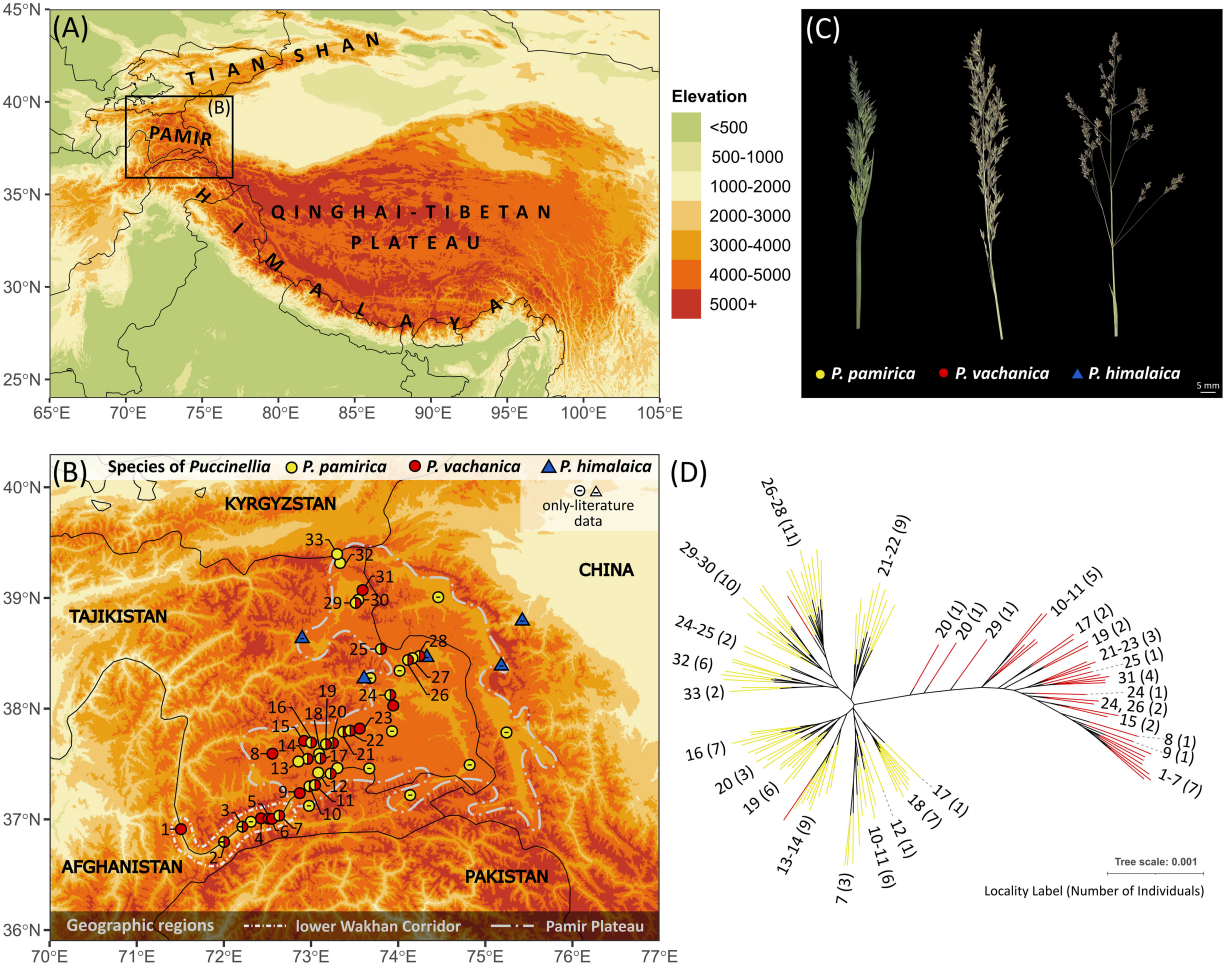
Distribution and spatial genetic structure of *Puccinellia pamirica* and its hybrids with *P. himalaica* (identified as *P. vachanica*) across the Pamir Mountains, Central Asia. **(A)** Geographic location of the research area in High Mountain Asia. Country borders are outlined in black. **(B)** Current distribution range of *P. pamirica*, *P. himalaica* and their hybrids (corresponding to the morphological description of *P. vachanica*) in the research area. Symbols with two colors on the distribution map represent occurrence of two taxa in the same locality. The position of labels representing closely located populations (15, 16, 19–22, 26–28) was slightly adjusted to ensure better clarity (full list of localities with their geographic coordinates is available in **Supplementary Table 2**). The colors used to represent a particular elevation range are the same as in **(A)**. The division between the lower Wakhan Corridor (the upper Panj River valley) and the Pamir Plateau represents the key landscape border, which divides these two ecologically different regions in the field. **(C)** Panicle morphology of *P. pamirica*, *P. vachanica*, and *P. himalaica* (see **Supplementary Figure 2** to compare panicle morphology of all alpine species occurring in the Pamir Mountains). **(D)** Spatial genetic structure of *P. pamirica* and *P. vachanica* across the Pamir Mountains inferred using maximum-likelihood in IQ-TREE ver. 1.6.12 based on 11,519 loci generated by the Diversity Arrays Technology sequencing (DArTseq) approach. A particular clade was preserved in the tree topology when it has the Shimodaira–Hasegawa-like approximate likelihood ratio test (SH-aLRT) value reaching at least 80% or the Ultrafast Bootstrap Approximation (UFBoot) value of at least 95%. Numbers of localities are given according to **(B)**.

The populations of *P. pamirica* and *P. himalaica* detected since the 20th century do not overlap with the current distribution range of *P. vachanica* (**Figures 5A, B**). *Puccinellia pamirica* occurs throughout the Pamir Mountains in all major patches of hypersaline wetland vegetation, mostly between 3,500 and 4,000 m a.s.l. The species is especially frequent on the Pamir Plateau, covering almost the entire latitudinal and longitudinal range of this region. Outside the plateau, *P. pamirica* was observed in isolated localities along the upper Panj River valley in the lower Wakhan Corridor, below 3,000 m a.s.l. In that region, we have found the species only in one place (Langar), which is the easternmost and highest elevated locality previously reported from the Panj River valley. Surprisingly, we did not observe any existing populations of *P. himalaica* in the Pamir Mountains despite extensive field research. Five localities noted in this region were derived from at least 20-year-old observations and a verified herbarium material older than 60 years (**Supplementary Table 2**).

Hybrids between *P. himalaica* and *P. pamirica* (all identified as *P. vachanica*) occurred abundantly in 28 localities, including 21 sites scattered throughout hypersaline wetlands on the Pamir Plateau and 7 established in saline enclaves along the lower Wakhan Corridor (**Figure 5B**). Almost half of these localities hosted both *P. pamirica* and hybrid individuals, along with the inherent absence of *P. himalaica*. Moreover, the hybrids occurred abundantly below 3,000 m a.s.l., in the upper Panj River valley, while both parental species remained undetected in these lowermost localities (**Supplementary Table 2**).

### 3.6 Spatial genetic structure

*Puccinellia pamirica* and its hybrids with *P. himalaica* (attributed to the morphotype of *P. vachanica*) demonstrated detectable genetic variation across the Pamir Mountains (**Figure 5**). *Puccinellia pamirica* represented two main genetic groups, corresponding to the southern and northern geographic groups of the species distribution range. Moreover, the genetic similarity among the individuals of *P. pamirica* mainly followed their spatial arrangement in the mountain valley system established on the Pamir Plateau and in the lower Wakhan Corridor. The individuals of *P. vachanica* resembled in part these distinct spatial genetic patterns detected in *P. pamirica*. However, the evolutionary relationships reconstructed in *P. pamirica* did not fully align with those revealed among hybrid individuals (**Figure 5**). This discrepancy could be the effect of a large difference in the number of sampled individuals between *P. pamirica* and *P. vachanica* (81 vs 36) or the genetic input of *P. himalaica*. Unfortunately, we were unable to examine intraspecific variation of *P. himalaica* throughout the Pamir Mountains due to the limited availability of plant material from the studied area (only one herbarium specimen was successfully genotyped from the Western Pshart River valley). Nevertheless, two individuals of *P. vachanica* (1395-1, 1399-4) were nested within a particular geographic clade of *P. pamirica*, reflecting their shared location-specific genetic footprints.

### 3.7 Lineage diversification and hybrid origin scenarios of *Puccinellia vachanica*

**Figure 6:**
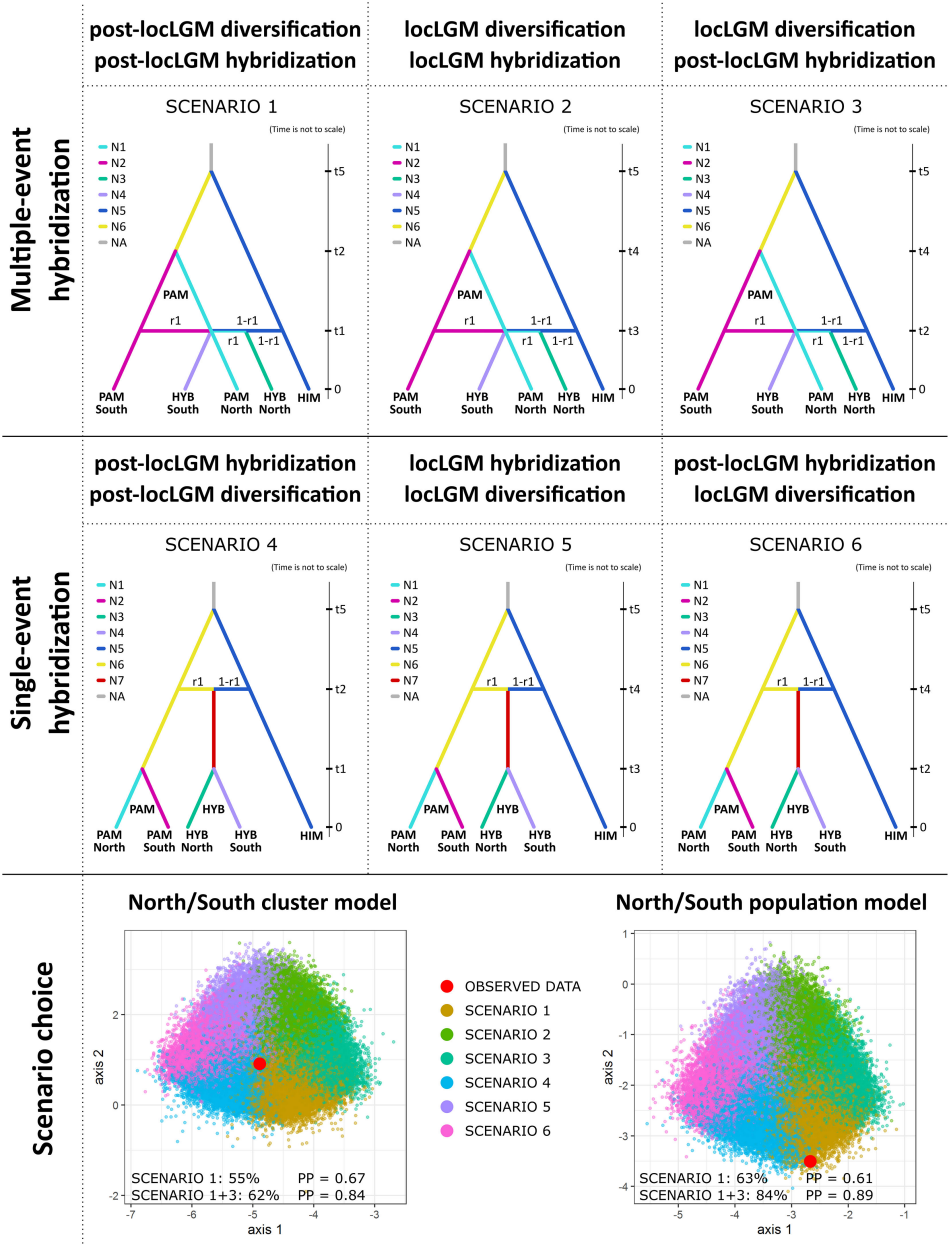
Lineage diversification history and scenarios on the origins of hybrids between *Puccinellia pamirica* and *P. himalaica* (identified as *P. vachanica*), tested using approximate Bayesian computation with supervised machine learning in DIYABC Random Forest ver. 1.0 based on the DArTseq SNP markers. Abbreviations: local Last Glacial Maximum in the Pamir Mountains at ~50,000–100,000 years ago (locLGM), interspecific gene flow between *P. pamirica* and *P. himalaica* (hybridization), intraspecific divergence of *P. pamirica* in multi-event hybridization scenarios or independent population divergence in *P. pamirica* and hybrid lineage in single-event hybridization scenarios (diversification), *P. pamirica* (PAM), *P. himalaica* (HIM), hybrids (HYB), effective population size of a lineage (N), time of a past event (t), admixture parameter (r_1_), posterior probability of the scenario choice (PP). Prior distributions of parameters: between 10 and 25,000 generations for t_1_ and t_2_ to represent the events after the local LGM (t_1_ < t_2_ when used together in a scenario); between 25,000 and 50,000 generations to represent the events during the local LGM (t_3_ and t_4_ parameters; t_3_ < t_4_ when used together in a scenario); between 100,000 and 200,000 generations to represent a split of the ancestral lineages of the parental species (t_5_ parameter). We assumed a 2-year generation time. The admixture rate derived from *P. pamirica* during interspecific hybridization with *P. himalaica* (r_1_ parameter) was set between 0.01 and 0.99. The North/South cluster model was based on 118 individuals divided according to their geographic location in the Pamir Mountains. The North/South population model was based on 27 individuals from two selected regions located either in the southern or northern part of the Pamir Mountains. Full results on the scenario choice from the DIYABC-RF analysis are presented in **Supplementary Tables 5**, **6**, **11**, **12**.

**Figure 7:**
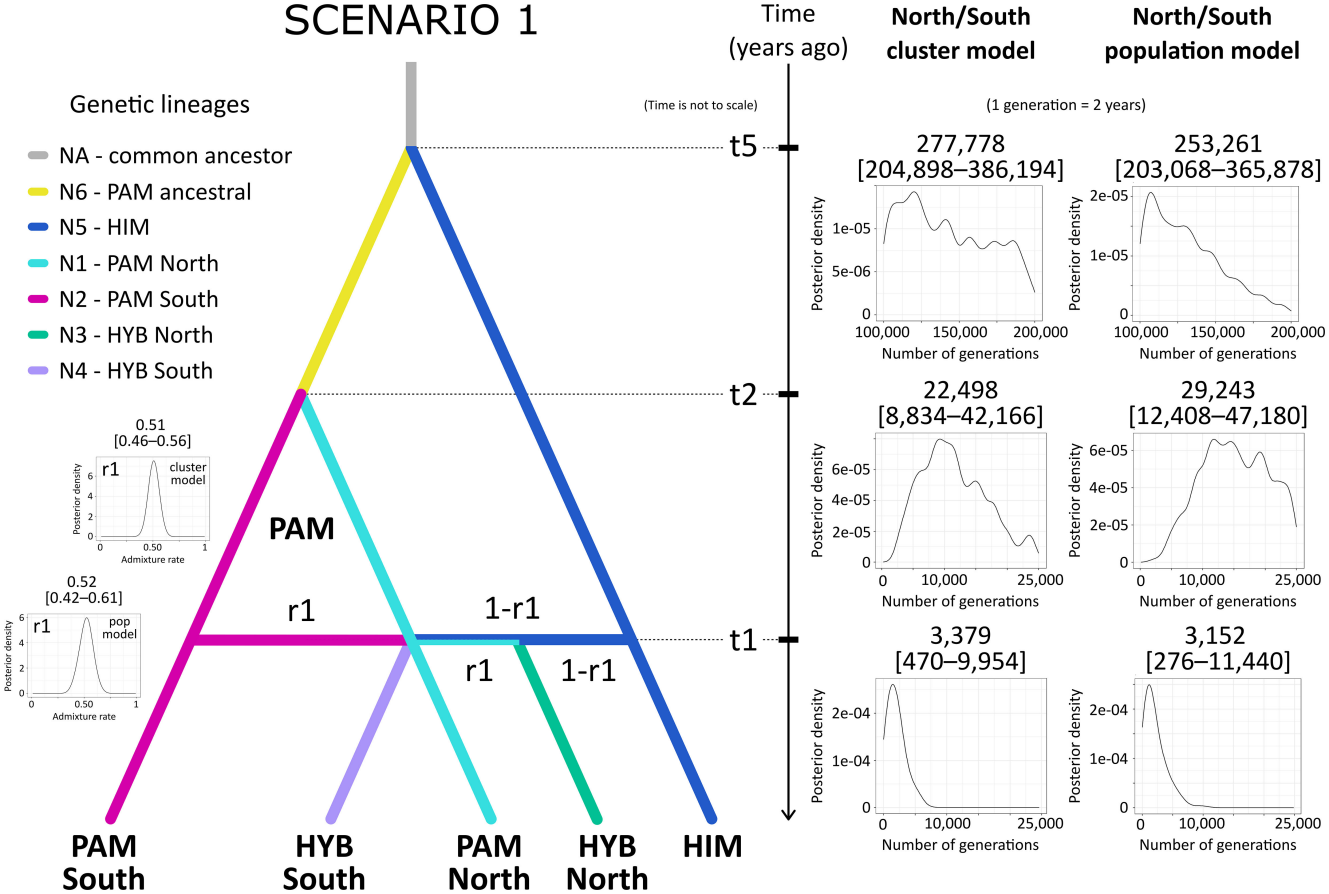
Parameter value estimation in the best supported scenario (Scenario 1) on the hybrid origins between *Puccinellia pamirica* and *P. himalaica* (identified as *P. vachanica*), performed using approximate Bayesian computation with supervised machine learning in DIYABC Random Forest ver. 1.0 based on the DArTseq SNP markers. Scenario 1 represents intraspecific diversification of *P. pamirica* after local Last Glacial Maximum in the Pamir Mountains at ~50,000–100,000 years ago followed by multiple independent hybridization events with *P. himalaica*. Median values with 5% and 95% quantiles are presented for the estimated model parameters. Abbreviations: *P. pamirica* (PAM), *P. himalaica* (HIM), hybrids (HYB), effective population size of a lineage (N), time of a past event (t), admixture parameter (r_1_). Prior distributions of parameters: between 10 and 25,000 generations for t_1_ and t_2_ to represent the events after the local LGM (t_1_ < t_2_ when used together in a scenario); between 100,000 and 200,000 generations to represent a split of the ancestral lineages of the parental species (t_5_ parameter). We assumed a 2-year generation time. The admixture rate derived from *P. pamirica* during interspecific hybridization with *P. himalaica* (r_1_ parameter) was set between 0.01 and 0.99. The North/South cluster model (cluster model) was based on 118 individuals divided according to their geographic location in the Pamir Mountains. The North/South population model (pop model) was based on 27 individuals from two selected regions located either in the southern or northern part of the Pamir Mountains. Full results on parameter value estimates from the DIYABC-RF analysis are presented in **Supplementary Tables 7** –**10**.

The ABC for population history inference supported multiple gene flow events between *P. pamirica* and *P. himalaica* after the local LGM in the Pamir Mountains as the best evolutionary scenario explaining the origins of their hybrids. Both models consistently determined that Scenario 1 is the best fitted option for the observed genetic data (**Figure 6**), gathering 55.2 ± 1.3% of the decision tree votes with posterior probability of 0.673 ± 0.022 in the North/South cluster model (**Supplementary Table 5**) and 63.1 ± 1.9% of the decision tree votes with posterior probability of 0.612 ± 0.019 in the North/South population model (**Supplementary Table 6**). According to this evolutionary scheme, both parental species hybridized independently in distant localities during the Holocene between ~300–11,000 years ago (t_1_ in **Figure 7** and in **Supplementary Table 7**), maintaining an almost uniform input of genetic admixture of ~0.50 (r_1_ in **Figure 4** and in **Supplementary Table 8**). We found no relevant support for scenarios that included a single-event formation of a hybrid lineage, which later developed its spatial genetic structure independent of *P. pamirica* and *P. himalaica* (Scenarios 4–6 in **Supplementary Tables 5** , **6**). Instead, hybrids inherited their fine-scale genetic patterns from their parental species. Most likely, the intraspecific genetic structure of *P. pamirica* was shaped after the local LGM, between ~11,000–45,000 years ago, as determined in Scenario 1 (t_2_ in **Figure 7** and in **Supplementary Table 9**). Nevertheless, the ABC models also indicated that there is some chance that the northern and southern lineages of *P. pamirica* diverged earlier, during the local LGM in the Pamir Mountains, according to the second best scenario (Scenario 3: 20.4 ± 1.1% of the decision trees voting for this scenario in the North/South cluster model and 29.9 ± 1.8% in the North/South population model; **Supplementary Tables 5**, **6**). Despite these differences, the timing of hybridization events between *P. pamirica* and *P. himalaica* was reconstructed similarly in both Scenario 1 and Scenario 3 and aligned with the Holocene (compare **Supplementary Tables 7**, **10**). The split of the ancestral lineages of the parental species remains on a deep evolutionary scale along the history of the taxa studied, and its time estimates (t_5_) could be underestimated due to a restricted sample size for *P. himalaica* (1 individual) and the assumed priors for t_5_.

Both Scenario 1 and Scenario 3 supported multiple-event origins of hybrids between *P. pamirica* and *P. himalaica* during the Holocene. When combined together, these two scenarios collected an overwhelming share of votes from the decision trees in the RF classifier, 62.0 ± 1.5% in the North/South cluster model (**Supplementary Table 11**) and 84.2 ± 1.4% in the North/South population model (**Supplementary Table 12**). Such scenario choice reached high posterior probability of 0.837 ± 0.027 and 0.892± 0.012 respectively (**Supplementary Tables 11**, **12**). These results show that *P. pamirica* first developed major patterns of its fine-scale genetic structure during the Late Pleistocene. Subsequently, the species passed its genetic variation on to interspecific crosses during multiple independent hybridization events with *P. himalaica* which occurred over the Holocene.

## 4 Discussion

Hybridization is an essential mechanism that affects genetic diversity and the process of speciation in flowering plants. In this study, we provide the first molecular evidence of reticulate evolution among alpine alkali grasses (Poaceae: *Puccinellia*). Genomic data coupled with morphological examination support the hypothesis that *P. vachanica* is an interspecific hybrid that emerged from two other alpine species occurring in High Mountain Asia, *P. pamirica* and *P. himalaica*.

### 4.1 Evolutionary history in a mixed-ploidy system

High-resolution phylogenies are crucial to resolve alpine plant radiations and uncover diversification trajectories of mountain species (Hughes and Atchison, 2015; Slovák et al., 2023; Carruthers et al., 2024). Despite recent technological advances, many species-rich plant groups still lack thorough genomic studies, which could shed more light on their infrageneric classification, microevolutionary processes, and drivers of speciation. The evolutionary history is particularly difficult to decipher when polyploidization has been involved alongside lineage formation, as frequently observed in flowering plants (Rothfels, 2021). Unlike diploids, polyploids possess multiple copies of a whole genome, which may complicate data analysis and confuse interpretation of the obtained results.

Alkali grasses are known for their wide array of ploidy levels (Kúr et al., 2023). However, alpine species are represented primarily by diploids and tetraploids according to a few available chromosome counts (**Supplementary Table 1**). Although ploidy levels could be estimated from genotyping-by-sequencing (GBS) data based on allelic ratios (Gompert and Mock, 2017; Wang et al., 2022), this approach was not applicable for our DArTseq dataset. In this study, only one homologous sequence was selected for each locus during complexity reduction for the examined *Puccinellia* species so that polyploid genotypes were subsampled to maintain a biallelic format. The subsampling procedure inherently introduced some information loss in our genomic SNP dataset, excluding the possibility of recovering full polyploid genotypes or to estimate ploidy levels from the GBS data. However, the applied procedure made polyploids equivalent to diploids, which is a prerequisite in some of the analyses used in this study, such as fastSTRUCTURE or TreeMix (Pickrell and Pritchard, 2012; Raj et al., 2014; Monnahan et al., 2019; Stift et al., 2019). The lack of subgenome phasing blends different evolutionary histories in genomic data, which can distort subsequent phylogenetic inference (Rothfels, 2021). In a bifurcating tree topology, polyploids and hybrids are usually positioned near a diploid progenitor, which represents one of their subgenomes (Dauphin et al., 2018). That effect is particularly well-pronounced for *P. vachanica*, which was resolved near its diploid parental species, *P. pamirica*, in the DArTseq-based phylogeny. Taking into account the large congruence among the genomic relationships and morphological discrimination, the subsampled DArTseq SNP panel still appeared sufficient to reconstruct key relationships among the examined mixed-ploidy alkali grasses. This is further supported by the haploid plastid data, which recovered the same key points of divergence in the tree topology as observed in the DArTseq-based phylogeny. Integrative evidence shows that our genomic SNP inference most likely captures the essence of the species genomic relationships, particularly in case of resolving the main phylogenetic lineages of the studied alkali grasses.

It is worth noting that the haploid plastid sequences used in this study proved useful for phylogenetic purposes in our mixed-ploidy system. Sequence conservatism and a shortage of polymorphic sites are often a serious constraint for plastid-based phylogenetics and single-locus DNA barcoding in plants (Krawczyk et al., 2018). Contrary to many other plant genera, even a few chloroplast markers applied in alkali grasses so far provided satisfactory resolution to differentiate at least some evolutionary lineages among the non-alpine species (Consaul et al., 2010; Soreng et al., 2022). We showed that even two combined chloroplast markers are sufficient to distinguish major evolutionary lineages and selected species among alpine alkali grasses. In addition, the chloroplast markers appeared relevant in determining the direction of reticulate processes, particularly when more distant species are involved, such as *P. pamirica* and *P. himalaica*. Future studies could further harness the potential of the plastome data, using the complete chloroplast genome as a super-barcode to evaluate infrageneric classification in *Puccinellia*.

### 4.2 Origins of *Puccinellia vachanica*

Integrative evidence indicates that *P. vachanica* emerged as an interspecific hybrid between *P. pamirica* and *P. himalaica*. These two species of *Pucccinellia* were consistently determined to be involved in the gene flow process using different analytical methods and subsetting schemes. Population clustering approaches could encounter difficulties in identifying hybrids that show clearly asymmetric parental contributions (Kong and Kubatko, 2021). According to our genomic SNP data, *P. vachanica* displayed almost symmetric ancestry between both parental species, and this pattern was successfully recovered by fastSTRUCTURE, STRUCTURE and TreeMix. This result was further supported by the ABC for population history inference, which explored a full range of possible admixture rates of *P. pamirica* and *P. himalaica* and still reconstructed ~0.5 genetic input from both progenitors in *P. vachanica*.

Chloroplast markers provided enough resolution to uncover a bidirectional gene flow between *P. pamirica* and *P. himalaica* in the Pamir Mountains. The plastid data itself show that hybridization must have occurred at least twice between these more distantly related alpine alkali grasses. The potential evolutionary scenarios of historical diversification in alpine species could be evaluated within the hypothesis-testing framework of comparative phylogeography (Papadopoulou and Knowles, 2016; Larsson et al., 2022). The distribution ranges of both *P. pamirica* and *P. himalaica* are not limited to the Pamir Mountains, covering other adjacent regions of High Mountain Asia as well (Ovchinnikov and Chukavina, 1957; Dickoré, 1995; Liang et al., 2006). Therefore, these two alpine alkali grasses could theoretically have given rise to *P. vachanica* outside our research area and colonized the Pamir Mountains later on. The key to unraveling the origins of *P. vachanica* lies in explaining the emergence of its spatial genetic structure observed across the Pamir Mountains, which resemble genetic patterns detected in *P. pamirica*. Isolation-by-distance has strongly shaped genetic differentiation in *P. pamirica*, whose current localities demonstrate distinct genetic footprints detectable by DArTseq markers even at a distance of a few kilometers (Wróbel et al., 2023). Therefore, the putative emergence of *P. vachanica* outside the Pamir Mountains would require the development of its fine-scale genetic patterns independently of *P. pamirica*, most likely during the potential colonization of the studied area from the external source. This hypothesis was not supported by the ABC results for population history inference (Scenarios 4–6). Instead, this analysis determined a much higher probability of the scenario in which hybrids inherited their fine-scale genetic structure from the progenitors, which had developed such genetic footprints earlier. According to this scenario, the parental species acquired their spatial patterns of genetic variation during the Late Pleistocene and hybridized independently in multiple localities across the Pamir Mountains over the Holocene. This evidence implies that both *Puccinellia* species probably grew in the same localities or at least in their close neighborhoods during their frequent gene flow events. However, the currently known distribution range of *P. himalaica* remains very restricted throughout the Pamir Mountains and hardly overlaps with the discovered populations of its hybrid descendant, *P. vachanica*.

### 4.3 What happened to *Puccinellia himalaica* in the Pamir Mountains?

#### 4.3.1 Possibility 1 – incomplete distribution data

Field observations and collected specimens validate the occurrence of species in a particular area. However, the lack of such records does not necessarily prove the absence of the species (Whittaker et al., 2005). Therefore, there is still a possibility that *P. himalaica* may remain undiscovered in the Pamir Mountains.

The Pamir landscape provides more potentially suitable locations for grassland species such as *P. himalaica* than it actually occupies according to the available field records. The species grows in similar moist habitats in neighboring Central Asian regions (Liang et al., 2006) and, as such, could demonstrate at least theoretical capacity to inhabit more patches of wetland-dependent vegetation currently established across the Pamir Mountains (Wróbel et al., 2023). This suggests that *P. himalaica* could be a part of dark diversity in the Pamir Mountains and still belong to the regional species pool of alpine grasslands despite its rare detectability in the field (Pärtel et al., 2011; Cornell and Harrison, 2014).

Based on data from the literature and revised herbarium materials, we determined that *P. himalaica* has been observed only in five localities in the Pamir Mountains. Herbarium collections provide an invaluable data source for evolutionary biology and global change research (Lavoie, 2013; Lang et al., 2019; Albani Rocchetti et al., 2021). Owing to the availability of the preserved specimens, we were able to acquire *P. himalaica* genotype from the Pamir Mountains, which is now the oldest verified record of the species in this alpine region (16 July 1958, Tzvelev, originally identified as *P. tenuiflora*; LE herbarium). In contrast, both *P. pamirica* and *P. vachanica* still occur frequently in the field and are scattered in multiple localities throughout the Pamir Plateau and the lower Wakhan Corridor. Despite the scarcity of available records of *P. himalaica*, it appears highly unlikely that this species has been constantly overlooked by researchers exploring these two Central Asian mountain regions so far. Although the identification of *Puccinellia* is often challenging, *P. himalaica* with its diffuse and smooth panicle, tiny glabrous lemmas, and very small anthers is morphologically distinct among other species occurring in Central Asia (Tzvelev, 1968; Dickoré, 1995; Liang et al., 2006). Therefore, this case does not resemble delayed discoveries of hardly recognizable cryptic species that remained undetected within a particular area for a long time (Bickford et al., 2007). Instead, previous researchers most likely did not observe or/and did not collect *P. himalaica* in the Pamir Mountains, rather than consistently confused it with other species.

During our field research in the Pamir Mountains, we frequently observed *Puccinellia* species noted in this mountain range earlier, however, with the striking exception of *P. himalaica*. All alpine species of this grass genus bloom abundantly over the short vegetation season in the summer months, particularly in July, and do not differ in phenology (Liang et al., 2006). Moreover, challenging identification of the genus in the field forces researchers to collect extensive plant material for further detailed laboratory examination, a situation that promotes detection of all species in a given area. In our opinion, the unconfirmed occurrence of *P. himalaica* in the lower Wakhan Corridor and its rarity throughout the Pamir Plateau may hardly be explained by the putative shortage of distribution data collected in these areas. Rather, we incline to the view that a few available contemporary records of *P. himalaica* suggest prevalent absence of the species in these geographic regions.

#### 4.3.2 Possibility 2 – warming-driven range shift of *Puccinellia himalaica*

We showed that gene flow between *P. himalaica* and *P. pamirica* was not limited to a single event and a mere coincidence, but rather occurred frequently throughout the Pamir Mountains. Moreover, the analyses revealed that hybrids most likely inherited their genetic variation from their progenitors that resided within a particular locality during gene flow events over the Holocene. Interspecific offspring can derive their fine-scale genetic structure from parental species alongside multiple independent hybridization events (Zalewska-Gałosz et al., 2023). Moreover, frequent gene flow could especially occur among species that demonstrate an overlap between their distribution ranges and share similar ecological niches (Gramlich et al., 2018; Baiakhmetov et al., 2021; Buono et al., 2021; Wu et al., 2022b; Sinaga et al., 2024). The lack of spatial barriers facilitates reciprocal exposure to gametes and may promote mating between species that have not yet developed reproductive barriers. Therefore, the hypothetical scenario of a former wider distribution range and a recent retreat of *P. himalaica* from the lower Wakhan Corridor and the Pamir Plateau could explain its current rarity across these alpine regions.

Upon global temperature rise, alpine plants demonstrate a general tendency for upslope distribution shifts (Elsen and Tingley, 2015; Liang et al., 2018; Vintsek et al., 2022). Therefore, *P. himalaica* might have retreated from the lower Wakhan Corridor and the Pamir Plateau before much extensive research began in these Central Asian regions. This could justify why there are only a few contemporary observations of this species from the Pamir Mountains gathered since the 20th century. Moreover, it is worth noting that the majority of available records of the genus *Puccinellia* collected in this region are restricted to the lower Wakhan Corridor and the Pamir Plateau, which are the easiest areas to explore across the Pamir Mountains (Ovchinnikov and Chukavina, 1957; Dickoré, 1995). Therefore, there is a chance that *P. himalaica* remains hidden in less penetrated and higher elevated regions, including vicinity of glacier foreheads, their run-off trails, and hardly accessible alpine valleys above 4,000 m a.s.l. Even if that is the case, the species would remain strongly pushed towards the upper limits of plant survival capacity in the Pamir Mountains.

Abrupt landscape changes and aggravating abiotic stress could become intolerable for insufficiently adapted species (Dullinger et al., 2012). *Puccinellia himalaica* is a component of moist alpine grasslands, including lake shores and glacier run-off paths. It is also one of a few representatives among extreme high-elevation grasses which could currently grow near and even above 5,000 m a.s.l. in High Mountain Asia (Dickoré, 1995; Liang et al., 2006). Although the species could tolerate increased soil salinity, it is not limited to the hypersaline grasslands, as observed for highly-specialized *P. pamirica* and the discovered hybrids. Arguably, *P. himalaica* could have experienced strong ecological turbulences over the Holocene due to its potential warming sensitivity and/or low competitiveness alongside changing environmental conditions in the Pamir Mountains (Wróbel et al., 2023). A pure lineage of *P. himalaica* could have been strongly limited by potentially unfavorable local species interactions, dispersal barriers or other ecological filters, leading to its regional extinction in the lower Wakhan Corridor and across the Pamir Plateau.

Hybrids or introgressed individuals could demonstrate a better adaptive potential than one or both of their parental species (Monnahan et al., 2019; Hodel et al., 2022). It appears that the crosses between *P. himalaica* and *P. pamirica* inherited the ability to resist hypersaline conditions considering their distribution range that largely overlaps with the localities occupied by the extremophilic halophyte *P. pamirica*. Furthermore, the persistence of hybrids in the lowermost and warmest localities of the studied area could suggest that a new genomic combination possibly provides a better capacity to tolerate higher summer temperatures than that shown by each parental lineage. This hypothesis would require further experimental confirmation. Hybridization may increase the adaptive potential and prevent extinction of the warming-sensitive species through evolutionary rescue (Charles and Stehlik, 2021; Brauer et al., 2023; Hansen, 2023). Therefore, the natural hybrids between *P. pamirica* and *P. himalaica* could potentially serve as a valuable reservoir to preserve the genetic material of their progenitors as temperature rise progresses.

#### 4.3.3 Possibility 3 – extinction via hybridization and decline of *Puccinellia himalaica*

Hybridization processes could eliminate one or both parental species by undermining their genetic integrity via introgression or impeding successful propagation of pure parental lines (Todesco et al., 2016). Due to the formation of interspecific hybrids with *P. pamirica*, the wasted reproductive effort of *P. himalaica* could have greatly decreased its chances of prolonging the species’ own distinct lineage, ultimately leading to its regional extinction. It is worth noting that all but one of the collected hybrids shared almost equal genetic input from both parental species, suggesting either F1 offspring or later crosses between hybrids. Similarly, hybrids represent intermediate morphological features between *P. pamirica* and *P. himalaica* in terms of panicle shape and color, as well as length of flower structures. We did not detect clinal genetic variation between *P. pamirica* and *P. himalaica*, which suggests the absence or marginal role of potential introgression in the studied group. This could imply sterility or general low fertility of the discovered hybrids, a common phenomenon among interspecific F1 offspring in flowering plants, or their reproductive incompatibility with the parental species (Steen et al., 2004; Cozzolino et al., 2006; Abbott, 2017). Previous cytological studies indicated that plants attributed to *P. vachanica* could be tetraploids (Sokolovskaya and Probatova, 1975). This may suggest that the diploid parental species *P. pamirica* possibly produces abundant and viable unreduced gametes, which are important components for successful reproduction in mixed-ploidy systems (Kolář et al., 2017). Therefore, determining ploidy levels and fertility among alpine species of *Puccinellia* from different geographic regions should be the next step in further evaluating the permeability of species boundaries and the consequences of gene flow events in these high-mountain plants (Buono et al., 2021).

Our results showed that most of the hybrid individuals originated after the merge of the paternal input of *P. pamirica* and maternal input of *P. himalaica.* Although our study design could not provide general quantification of the frequency of hybridization, it is notable that we collected much fewer individuals with the opposite direction of gene flow throughout the whole study area. This could imply that the pollen of diploid *P. pamirica* imposed a particularly strong pressure on the female gametes of tetraploid *P. himalaica* within the shared localities. Pervasive gene flow from diploids to tetraploids is frequently observed in flowering plants when different cytotypes occur in sympatry (Zohren et al., 2016; Monnahan et al., 2019). Past distribution shifts and similar habitat requirements of these two *Puccinellia* species could have led to their frequent range overlaps in the Pamir Mountains during the Late Quaternary climate oscillations (Muellner-Riehl, 2019; Wróbel et al., 2023). As such, intense interspecific hybridization might have prevented the successful propagation of pure *P. himalaica*, leading to its progressing decline in the vicinity of *P. pamirica* over the Holocene. Consequently, *P. himalaica* could have disappeared from numerous localities across the Pamir Plateau and the lower Wakhan Corridor, where the currently remaining hybrids preserve the genetic legacy of their already extinct parental species.

### 4.4 Conservation implications and taxonomic treatment

The alpine biome is susceptible to profound ecological changes under climate upheavals and is especially fragile to temperature rise. Upon global warming, mountain ecosystems could be particularly prone to severe environmental changes and habitat transformations accompanied by dramatic species turnovers (Steinbauer et al., 2018). Deciphering how mountain flora was assembled and how alpine diversity responded to past climate changes may be the key to better forecast future impact of current temperature rise on high-elevation species (Wróbel et al., 2023; Carruthers et al., 2024; Kandziora et al., 2024). A solid understanding of biological processes in the alpine biome is essential to identify mechanisms responsible for potential ecological threats in various groups of cold-adapted species and across distinct geographic regions (Bellard et al., 2012). The contrasting outcomes of hybridization events urge us to reveal their consequences in shaping evolutionary patterns across different biological systems. In particular, special attention should be paid to the potentially negative effects of interspecific gene flow that could threaten the integrity or even existence of the involved parental lineages (Vallejo-Marin and Hiscock, 2016; Nobis et al., 2019). The extensive regional framework devoted to evolutionary processes would contribute to the development of effective management strategies and the selection of key conservation priorities across High Mountain Asia in view of the continuing global warming (Yu et al., 2019; Nobis et al., 2023).

Our results suggest that *P. himalaica* has possibly declined in the Pamir Mountains over the Holocene. Even ~300 years ago, the species could still have occurred in multiple localities currently occupied by its hybrids with *P. pamirica*. *Puccinellia himalaica* could have retreated from the Pamir Plateau and the lower Wakhan Corridor to the remote high-mountain refugia or already become extinct in numerous locations throughout these Central Asian regions. A highly restricted distribution range limited to five known and isolated populations qualify the species as critically endangered in the Pamir Mountains according to the IUCN criteria B2ab (IUCN, 2012). Such a regional threat category could even be too optimistic as all species populations were observed at least 20 years ago in this mountain range and since then have remained unconfirmed.

Our research shows that *P. vachanica* should be considered a natural hybrid between *P. pamirica* and *P. himalaica* with a poorly recognized distribution range in Central Asia and hereafter classified as the nothospecies *Puccinellia* ×*vachanica* Ovcz. & Czukav. (pro species). This implies that previous records related to *P. vachanica* could potentially be attributed to hybrids between *P. himalaica* and *P. pamirica* throughout High Mountain Asia. If that is the case, these interspecific crosses could also occur beyond the Pamir Plateau and the lower Wakhan Corridor. The putative occurrence of these hybrids could extend to the Qinghai, southern Xinjiang, and western Xizang (Tibet) provinces, where plants corresponding to the morphotype of *P. vachanica* were previously observed (Liang et al., 2006). Notably, such a geographic extent covers a substantial part of the known distribution ranges of both *P. pamirica* and *P. himalaica* (Dickoré, 1995; Liang et al., 2006). Interspecific hybridization can especially threaten rare taxa that are exposed to potential gene exchange with their more widespread or competitive relatives (Aerts et al., 2013; Macková et al., 2018). A wasted reproductive effort or a pressure from a genetic invasion via introgression could lead to a decline of a particular species, populations, or unique evolutionary variants, raising serious conservation concerns (Vilà et al., 2000; Todesco et al., 2016). Our results indicate that climate warming and interspecific hybridization could entail a high risk of extinction for *P. himalaica* in the wild. Therefore, there may be an urgent need to monitor the remaining pure populations of *P. himalaica* in High Mountain Asia and preserve the available genetic resources of this high-elevation specialist.

Alarming rates of current climate changes can trigger another wave of ecological turbulences, leading to major species turnovers in the alpine biome (Knight, 2022; Schuchardt et al., 2023). The unprecedented speed of environmental changes and the associated shifts of species distribution ranges could provide new opportunities for gene flow between species that were geographically but not reproductively isolated (Vallejo-Marin and Hiscock, 2016). In particular, contact zones may be established between cold-adapted specialists and their lowland counterparts, which could disperse upslope upon further temperature rise. There is concern that these warm-adapted and often more widespread newcomers could blur or even completely absorb gene pools of their alpine relatives (Seehausen, 2006; Gómez et al., 2015). Although hybridization could pose a threat to the genetic integrity of cold-adapted species, it could also become the solution to increase the adaptive potential of alpine species and preserve at least partially their genetic legacy in the future (Charles and Stehlik, 2021). Technological advances in genomics could nowadays provide important evidence to monitor biological processes and inform decision-making in biodiversity management (Schmidt et al., 2023; Theissinger et al., 2023). Therefore, the potential eco-evolutionary outcomes of interspecific hybridization should receive special attention in developing conservation strategies for cold-adapted species in view of progressing global warming.

## Data availability statement

The datasets presented in this study can be found in online repositories. The names of the repository/repositories and accession number(s) can be found below: https://www.ncbi.nlm.nih.gov/genbank/, PP457807 PP457808 PP457809 PP457810 PP457811 PP457812 PP457813 PP457814 PP457815 PP457816 PP457817 PP457818 PP457819 PP457820 PP457821 PP457822 PP457823 PP457824 PP457825 PP457826 PP457827 PP457828 PP457829 PP457830 PP457831 PP457832 PP457833 PP457834 PP457835 PP457836 PP457837 PP457838 PP457839 PP457840 PP457841 PP457842 PP457843 PP457844 PP457845 PP457846 PP457847 PP457848 PP457849 PP457850 PP457851 PP457852 PP457853 PP457854 PP457855 PP457856 PP457857 PP457858 PP457859 PP457860 PP457861 PP457862 PP457863 PP457864 PP457865 PP457866 PP457867 PP457868 PP457869 PP457870 PP457871 PP457872 PP457873 PP457874 PP457875 PP457876 PP457877 PP457878 PP457879 PP457880 PP457881 PP457882 PP457883 PP457884 PP457885 PP457886 PP457887 PP457888 PP457889 PP457890 PP457891 PP457892 PP457893 PP457894 PP457895 PP457896 PP457897 PP457898 PP457899 PP457900 PP457901 PP457902 PP457903 PP457904 PP457905 PP457906 PP457907 PP457908 PP457909 PP457910 PP457911 PP457912 PP457913 PP457914 PP457915 PP457916 PP457917 PP457918 PP457919 PP457920 PP457921 PP457922 PP457923 PP457924 PP457925 PP457926 PP457927 PP457928 PP457929 PP457930 PP457931 PP457932 PP457933 PP457934 PP457935 PP457936 PP457937 PP457938 PP457939 PP457940 PP457941 PP457942 PP457943 PP457944 PP457945 PP457946 PP457947 PP457948 PP457949 PP457950 https://doi.org/10.5281/zenodo.10793459.

## Author contributions

AW: Conceptualization, Formal Analysis, Funding acquisition, Investigation, Methodology, Project administration, Visualization, Writing – original draft, Writing – review & editing. EK: Investigation, Writing – review & editing. MN: Conceptualization, Investigation, Writing – review & editing.

## Supplementary material

Supplementary Figure 1 Selection of the genetic group number in STRUCTURE based on Evanno’s *ΔK* method.

Supplementary Figure 2 Panicle morphology of the examined alpine species of *Puccinellia.*

Supplementary Figure 3 Variation in the panicle morphology of *Puccinellia vachanica.*

Supplementary Table 1 Ploidy levels reported for the examined *Puccinellia* species according to the available published records.

Supplementary Table 2 List of the examined specimens of *Puccinellia* with the information on the sampled localities and GenBank accession numbers for chloroplast markers.

Supplementary Table 3 Morphological dataset of the examined specimens of *Puccinellia.*

Supplementary Table 4 Summary of the number of genetic group recovered across replicates runs in fastSTRUCTURE and STRUCTURE analyses.

Supplementary Table 5 Scenario choice in the North/South cluster model in DIYABC-RF analysis.

Supplementary Table 6 Scenario choice in the North/South population model in DIYABC-RF analysis.

Supplementary Table 7 Statistics of posterior distribution estimated for the model parameter t1 of Scenario 1 in DIYABC-RF analysis.

Supplementary Table 8 Statistics of posterior distribution estimated for the model parameter r1 of Scenario 1 in DIYABC-RF analysis.

Supplementary Table 9 Statistics of posterior distribution estimated for the model parameter Scenario 1 in DIYABC-RF analysis.

Supplementary Table 10 Statistics of posterior distribution estimated for the model parameter t2 of Scenario 3 in DIYABC-RF analysis.

Supplementary Table 11 Scenario choice with grouping in the North/South cluster model in DIYABC-RF analysis.

Supplementary Table 12 Scenario choice with grouping in the North/South population model in DIYABC-RF analysis.

Supplementary Presentation 1 SNP Marker discovery and genotyping using the DArTseq platform.
