# Supplementary figures and images for: Hybrids as mirrors of the past: genomic footprints reveal spatio-temporal dynamics and extinction risk of alpine extremophytes in the mountains of Central Asia
Source: Front Plant Sci. 2024 Apr 17;15:1369732. doi: 10.3389/fpls.2024.1369732 (PMC11061500; doi:10.3389/fpls.2024.1369732)

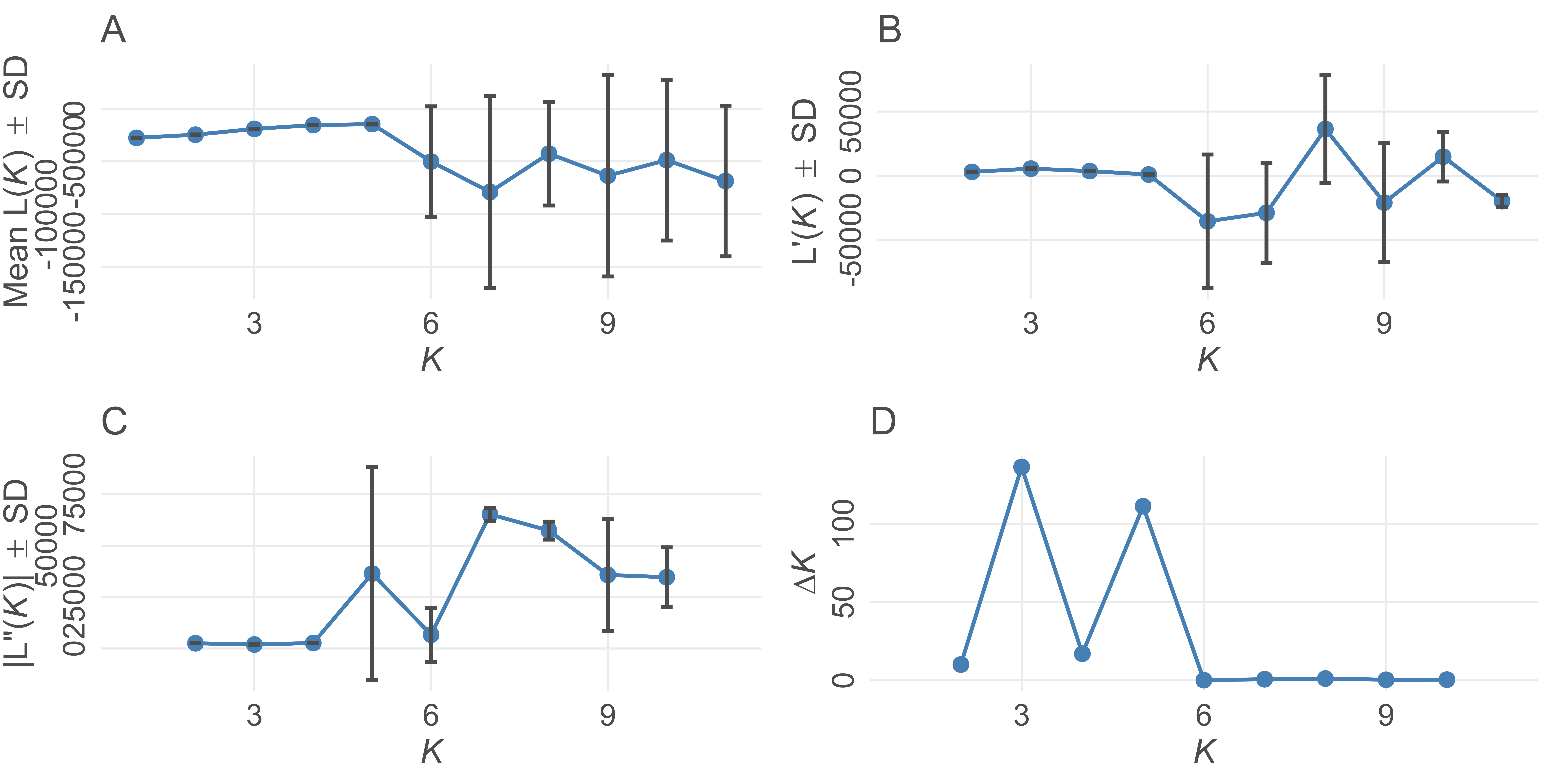

Supplement: Supplementary Figure 1 — Selection of the genetic group number in STRUCTURE based on Evanno’s ΔK method. [file Image_1.png]

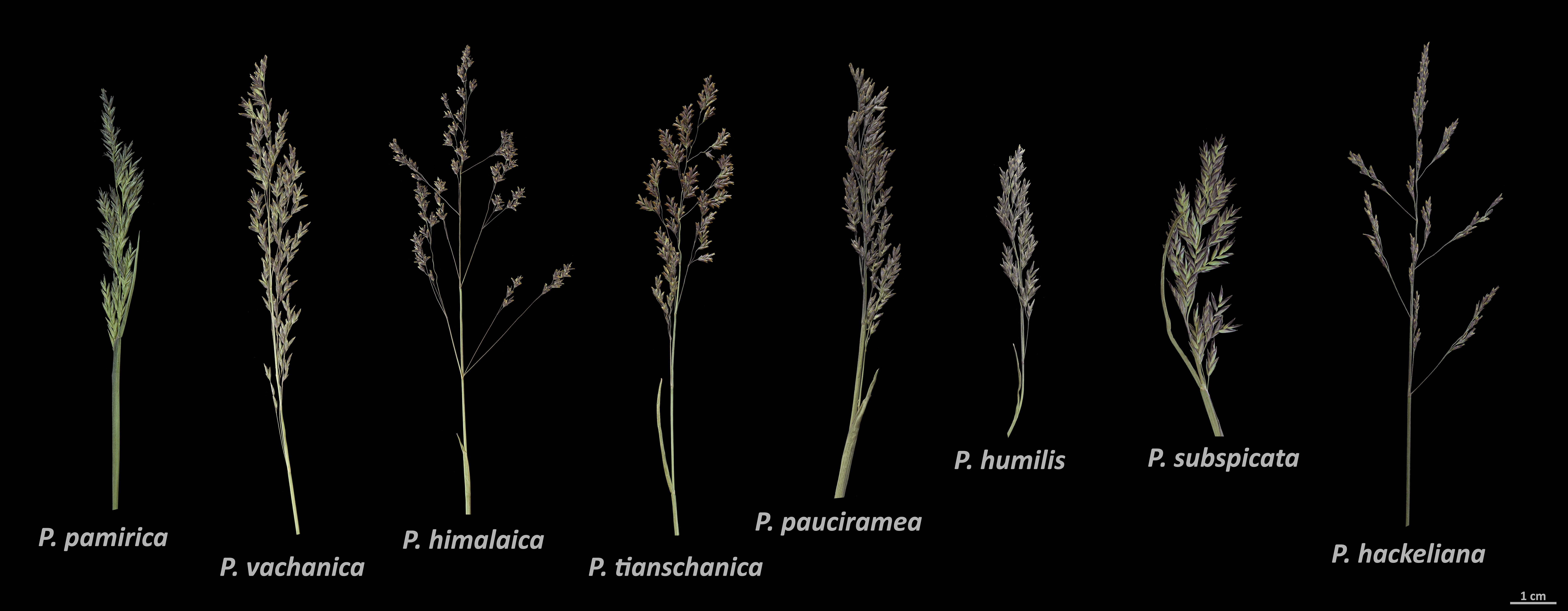

Supplement: Supplementary Figure 2 — Panicle morphology of the examined alpine species of Puccinellia. [file Image_2.jpeg]

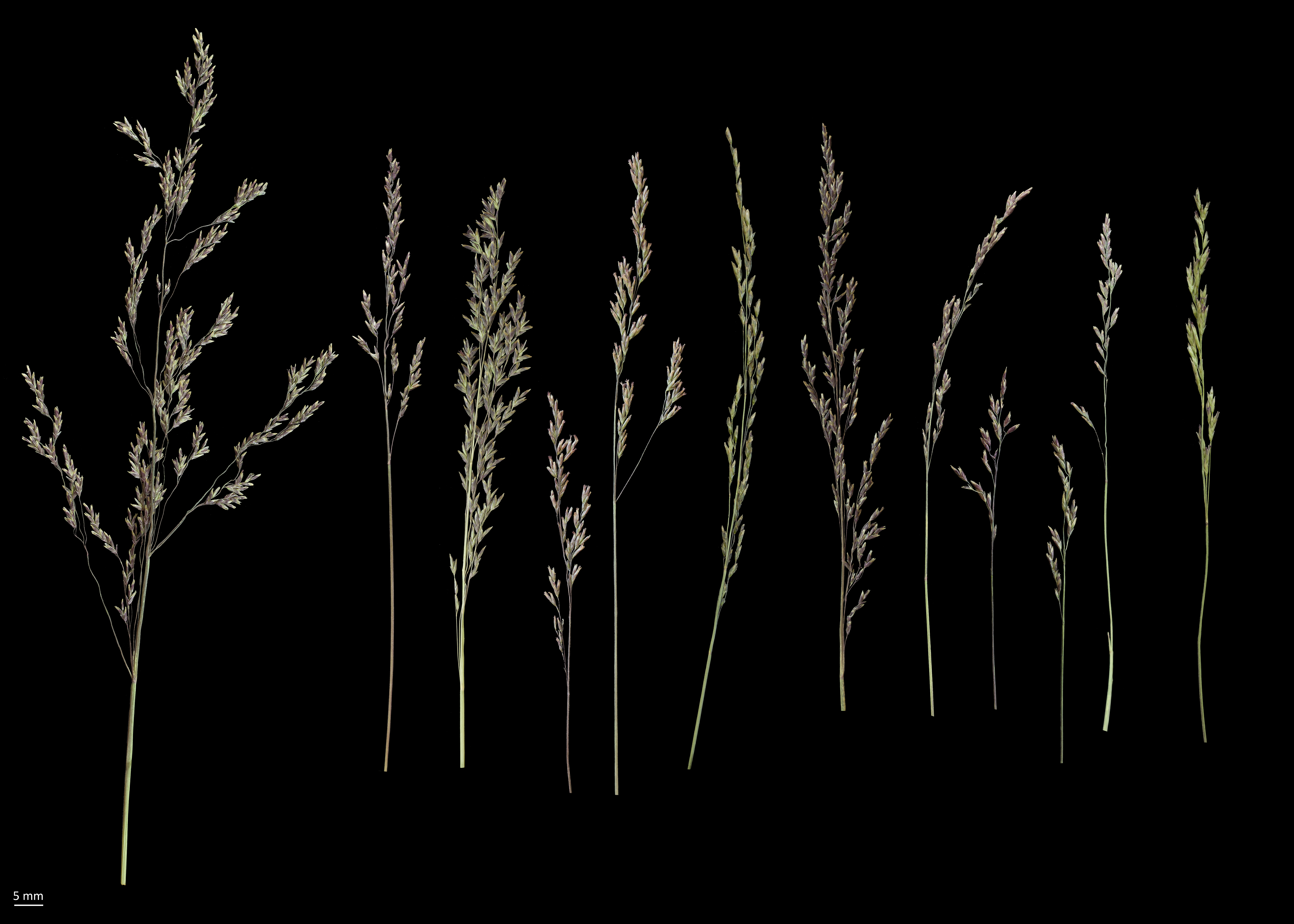

Supplement: Supplementary Figure 3 — Variation in the panicle morphology of Puccinellia vachanica. [file Image_3.jpeg]
