# Supplementary material for: Hybrids as mirrors of the past: genomic footprints reveal spatio-temporal dynamics and extinction risk of alpine extremophytes in the mountains of Central Asia
Source: Front Plant Sci. 2024 Apr 17;15:1369732. doi: 10.3389/fpls.2024.1369732 (PMC11061500; doi:10.3389/fpls.2024.1369732)
Supplement: Supplementary Table 1 — Ploidy levels reported for the examined Puccinellia species according to the available published records. [file Table_1.docx]

| Taxon | Ploidy | Chromosome counts reported in the reference | Reference |
| --- | --- | --- | --- |
| *P. distans* agg. | 4x, 6x; 2n = 28, 42 2x, 4x, 6x; 2n = 14, 28, 42 2x, 4x, 5x, 6x, 7x; 2n = 14, 28, 35, 42, 49 | 2n = 28, 42 2n = 14, 28, 42 2n = 14, 28, 35, 42, 49 | (Hughes, 1976) (Davis and Consaul, 2007)  (Kúr et al., 2023) |
| *P. glauca* (Regel) V.I.Krecz. [*P. distans* subsp. *glauca* (Regel) Tzvel.] | 6x; 2n =42 | 2n = 42 | (Tzvelev, 1976) |
| *P. hauptiana* (Trin. ex V.I.Krecz.) Kitagawa [*P. distans* subsp. *hauptiana* (Trin. ex V.I.Krecz.) W.E. Hughes] | 4x; 2n = 28 4x, 6x; 2n = 28, 42 | 2n = 28 2n = 28, 42 | (Tzvelev, 1976) (Hughes and Halliday, 1980) |
| *P. gigantea* (Grossh.) Grossh. | 2x; 2n = 14 | 2n = 14 | (Sokolovskaya and Probatova, 1975) |
| *P. hackeliana* (V.I.Krecz.) V.I.Krecz. ex Drobow | 4x; 2n = 28 6x; 2n =42 | 2n =28 2n = 42 | (Zakharyeva, 1985) (Tzvelev, 1976) |
| *P. himalaica* Tzvelev | 4x; 2n = 28 | n = 14 | (Kumar et al., 2018) |
| *P. humilis* (Litv. ex V.I.Krecz.) Bor | NA | NA | NA |
| *P. pamirica* (Roshev.) V.I.Krecz. ex Ovcz. & Czukav. | 2x; 2n = 14 | 2n = 14 | (Sokolovskaya and Probatova, 1975; Zakharjeva, 1993) |
| *P. pauciramea* (Hackel) V.I.Krecz. ex Ovcz. & Czukav. | 4x; 2n = 28 | 2n = 28 | (Sokolovskaya and Probatova, 1975) |
| *P. schischkinii* Tzvelev | 2x; 2n = 14 | 2n = 14 | (Probatova et al., 2013) |
| *P. subspicata* V.I.Krecz. ex Ovcz. & Czukav. | 2x; 2n = 14 | 2n = 14 | (Guinochet and Lefranc, 1981) |
| *P. tianschanica* (Tzvelev) Ikonn. | 2x; 2n = 14 | 2n = 14 | (Zakharjeva, 1993) |
| *P. tenuiflora* (Griseb.) Scribn. & Merr. | 2x; 2n = 14 4x; 2n = 28 | 2n = 14 2n = 28 | (Tzvelev, 1976) (Yan et al., 1989) |
| *P. vachanica* Ovcz. & Czukav. | 4x; 2n = 28 | 2n = 28 | (Sokolovskaya and Probatova, 1975) |

References

Davis, J. I., and Consaul, L. L. (2007). “Puccinellia Parl.,” in *Flora of North America North of Mexico 24: Magnoliophyta: Commelinidae (in part): Poaceae, part 1*, eds. M. E. Barkworth, K. M. Capels, S. Long, L. K. Anderton, and M. B. Piep (New York: Oxford University Press), 459–478.

Guinochet, M., and Lefranc, M. (1981). “Puccinellia subspicata V. Krecz.,” in *Chromosome Number Reports LXXIII*, ed. Á. Löve (Wiley), 853.

Hughes, W. ., and Halliday, G. (1980). “Puccinellia Parl.,” in *Flora Europaea 5*, eds. T. G. Tutin, V. H. Heywood, N. A. Burges, and D. H. Valentine (Cambridge: Cambridge University Press), 167–170.

Hughes, W. E. (1976). The taxonomy of the genus Puccinellia Parl. (Gramineae).

Kumar, R., Kumari, V., and Kumar Singhal, V. (2018). “Puccinellia himalaica Tzvelev,” in *IAPT chromosome data 28. Taxon*, eds. K. Marhold and J. Kučera (Wiley), 1239. doi: 10.12705/676.39.

Kúr, P., Gregor, T., Jandová, M., Mesterházy, A., Paule, J., Píšová, S., et al. (2023). Cryptic invasion suggested by a cytogeographic analysis of the halophytic Puccinellia distans complex (Poaceae) in Central Europe. *Front. Plant Sci.* 14, 1–10. doi: 10.3389/fpls.2023.1249292.

Probatova, N., Kazanovsky, S., Rudyka, E., Seledets, V., and Ovchinnikova, S. (2013). “Puccinellia schischkinii Tzvelev,” in *IAPT/IOPB chromosome data 16. Taxon*, eds. K. Marhold and I. Breitwieser (Wiley), 1360. doi: 10.12705/626.41.

Sokolovskaya, A. P., and Probatova, N. S. (1975). Chromosome numbers of some grasses (Poaceae) of the flora of USSR. *Bot. Zhurnal* 60, 667–678.

Tzvelev, N. N. (1976). *Zlaki SSSR*. Leningrad: Izdatelstvo Nauka.

Yan, G. x., Zhang, S. z., Yan, J. f., Fu, X. q., and Wang, L. y. (1989). Chromosome numbers and geographical distribution of 68 species of forage plants. *Grassl. China* 4, 53–60.

Zakharjeva, O. I. (1993). “Numeri Chromosomatum Magnoliophytorum Florae URSS, Moraceae–Zygophyllaceae,” in *Numeri Chromosomatum Magnoliophytorum Florae URSS, Moraceae–Zygophyllaceae*, ed. A. Takhtajan (Petropoli: Nauka).

Zakharyeva, O. I. (1985). Chromosome numbers of some flowering plants from the Caucasus and Middle Asia. *Bot. Zhurnal SSSR* 70, 1699–1701.
