# Supplementary material for: Hybrids as mirrors of the past: genomic footprints reveal spatio-temporal dynamics and extinction risk of alpine extremophytes in the mountains of Central Asia
Source: Front Plant Sci. 2024 Apr 17;15:1369732. doi: 10.3389/fpls.2024.1369732 (PMC11061500; doi:10.3389/fpls.2024.1369732)
Supplement: Supplementary Table 5 — Scenario choice in the North/South cluster model in DIYABC-RF analysis. [file Table_5.docx]

**Supplementary Table 5.** Lineage diversification history and scenarios on origins of *Puccinellia ×vachanica* emerging from *P. pamirica* and *P. himalaica*, tested by the approximate Bayesian computation with supervised machine learning in DIYABC-RF ver. 1.2.1. **Scenario choice** for each of the ten replicate analyses was based on 10 different reference tables comparing six tested scenarios in the **North/South cluster model** (118 individuals). For each reference table, the number of datasets simulated using DIYABC-RF was set to 10,000 per scenario and the number of RF-trees was 500. Scenarios are shown on **Figure 6**.

| **Reference table** | **Best scenario** | **Votes on scenario (proportion per 500 votes)** | | | | | | **Prior error rate** | **Posterior probability (best scenario)** |
| --- | --- | --- | --- | --- | --- | --- | --- | --- | --- |
|  |  |  |  |  |  |  |  |  |  |
|  |  | **1** | **2** | **3** | **4** | **5** | **6** |  |  |
| 1 | 1 | 0.560 | 0.006 | 0.206 | 0.148 | 0.010 | 0.070 | 0.236 | 0.698 |
| 2 | 1 | 0.560 | 0.006 | 0.196 | 0.168 | 0.012 | 0.058 | 0.236 | 0.669 |
| 3 | 1 | 0.518 | 0.010 | 0.224 | 0.192 | 0.004 | 0.052 | 0.235 | 0.707 |
| 4 | 1 | 0.548 | 0.012 | 0.196 | 0.192 | 0.012 | 0.060 | 0.234 | 0.654 |
| 5 | 1 | 0.546 | 0.004 | 0.200 | 0.172 | 0.008 | 0.070 | 0.235 | 0.665 |
| 6 | 1 | 0.554 | 0.006 | 0.196 | 0.184 | 0.012 | 0.048 | 0.235 | 0.668 |
| 7 | 1 | 0.558 | 0.004 | 0.218 | 0.166 | 0.010 | 0.044 | 0.235 | 0.665 |
| 8 | 1 | 0.546 | 0.006 | 0.212 | 0.170 | 0.006 | 0.060 | 0.235 | 0.644 |
| 9 | 1 | 0.564 | 0.008 | 0.188 | 0.158 | 0.006 | 0.076 | 0.234 | 0.705 |
| 10 | 1 | 0.566 | 0.008 | 0.204 | 0.160 | 0.010 | 0.052 | 0.235 | 0.651 |
| **Mean** | | 0.552 | 0.007 | 0.204 | 0.171 | 0.009 | 0.059 | 0.235 | 0.673 |
| **SD** | | 0.013 | 0.002 | 0.011 | 0.014 | 0.003 | 0.010 | 0.001 | 0.022 |
