# Supplementary material for: Hybrids as mirrors of the past: genomic footprints reveal spatio-temporal dynamics and extinction risk of alpine extremophytes in the mountains of Central Asia
Source: Front Plant Sci. 2024 Apr 17;15:1369732. doi: 10.3389/fpls.2024.1369732 (PMC11061500; doi:10.3389/fpls.2024.1369732)
Supplement: Supplementary Table 6 — Scenario choice in the North/South population model in DIYABC-RF analysis. [file Table_6.docx]

**Supplementary Table 6.** Lineage diversification history and scenarios on origins of *Puccinellia ×vachanica* emerging from *P. pamirica* and *P. himalaica*, tested by the approximate Bayesian computation with supervised machine learning in DIYABC-RF ver. 1.2.1. **Scenario choice** for each of the ten replicate analyses was based on 10 different reference tables comparing six tested scenarios in the **North/South population model** (27 individuals). For each reference table, the number of datasets simulated using DIYABC-RF was set to 10,000 per scenario and the number of RF-trees was 500. Scenarios are shown on **Figure 6**.

| **Reference table** | **Best scenario** | **Votes on scenario (proportion per 500 votes)** | | | | | | **Prior error rate** | **Posterior probability (best scenario)** |
| --- | --- | --- | --- | --- | --- | --- | --- | --- | --- |
|  |  |  |  |  |  |  |  |  |  |
|  |  | **1** | **2** | **3** | **4** | **5** | **6** |  |  |
| 1 | 1 | 0.662 | 0.014 | 0.264 | 0.050 | 0.004 | 0.006 | 0.276 | 0.617 |
| 2 | 1 | 0.616 | 0.004 | 0.316 | 0.054 | 0.006 | 0.004 | 0.274 | 0.589 |
| 3 | 1 | 0.644 | 0.008 | 0.294 | 0.052 | 0.000 | 0.002 | 0.276 | 0.606 |
| 4 | 1 | 0.614 | 0.006 | 0.320 | 0.052 | 0.006 | 0.002 | 0.275 | 0.624 |
| 5 | 1 | 0.642 | 0.010 | 0.294 | 0.054 | 0.000 | 0.000 | 0.275 | 0.655 |
| 6 | 1 | 0.610 | 0.010 | 0.304 | 0.072 | 0.002 | 0.002 | 0.275 | 0.600 |
| 7 | 1 | 0.636 | 0.002 | 0.296 | 0.062 | 0.000 | 0.004 | 0.276 | 0.632 |
| 8 | 1 | 0.640 | 0.004 | 0.292 | 0.054 | 0.002 | 0.008 | 0.275 | 0.608 |
| 9 | 1 | 0.598 | 0.006 | 0.330 | 0.054 | 0.006 | 0.006 | 0.275 | 0.601 |
| 10 | 1 | 0.646 | 0.008 | 0.280 | 0.060 | 0.000 | 0.006 | 0.276 | 0.591 |
| **Mean** | | 0.631 | 0.007 | 0.299 | 0.056 | 0.003 | 0.004 | 0.275 | 0.612 |
| **SD** | | 0.019 | 0.003 | 0.018 | 0.006 | 0.003 | 0.002 | 0.001 | 0.019 |
