# Supplementary material for: Hybrids as mirrors of the past: genomic footprints reveal spatio-temporal dynamics and extinction risk of alpine extremophytes in the mountains of Central Asia
Source: Front Plant Sci. 2024 Apr 17;15:1369732. doi: 10.3389/fpls.2024.1369732 (PMC11061500; doi:10.3389/fpls.2024.1369732)
Supplement: Supplementary Table 7 — Statistics of posterior distribution estimated for the model parameter t1 of Scenario 1 in DIYABC-RF analysis. [file Table_7.docx]

**Supplementary Table 7.** Estimations of posterior median as well as 5% and 95% quantiles (90% credibility interval) of the **parameter** **t_1_** (time of multiple post-local LGM interspecific hybridization events between *Puccinellia pamirica* and *P. himalaica*) performed using 10,000 simulations of the best supported scenario (**Scenario 1**) based on ten replicate analyses. The parameter **t_1_** was modeled using a prior distribution set between 10 and 25,000 generations (interpreted as a period after the local Last Glacial Maximum in the Pamir Mountains estimated between 50,000–100,000 years BP; we assumed 2-year generation time). The analysis was performed using the approximate Bayesian computation with supervised machine learning in DIYABC-RF ver. 1.2.1. Scenario 1 is shown on **Figure 6**.

| **North/South cluster model** | | | | **North/South population model** | | | |
| --- | --- | --- | --- | --- | --- | --- | --- |
| Reference table | Median | q5% | q95% | Reference table | Median | q5% | q95% |
| 1 | 1794 | 199 | 4955 | 1 | 1642 | 134 | 5814 |
| 2 | 1593 | 241 | 4679 | 2 | 1572 | 113 | 5791 |
| 3 | 1690 | 250 | 5059 | 3 | 1486 | 135 | 5709 |
| 4 | 1760 | 274 | 4783 | 4 | 1607 | 134 | 5701 |
| 5 | 1622 | 205 | 5190 | 5 | 1650 | 135 | 5834 |
| 6 | 1690 | 254 | 4987 | 6 | 1548 | 128 | 5836 |
| 7 | 1598 | 254 | 4817 | 7 | 1444 | 107 | 5502 |
| 8 | 1708 | 255 | 5234 | 8 | 1606 | 135 | 6049 |
| 9 | 1739 | 179 | 5114 | 9 | 1551 | 127 | 5239 |
| 10 | 1702 | 237 | 4950 | 10 | 1654 | 233 | 5727 |
| Mean [generations] | 1689 | 235 | 4977 | Mean [generations] | 1576 | 138 | 5720 |
| SD | 64 | 29 | 170 | SD | 67 | 33 | 207 |
| **Mean [years]** | **3379** | **470** | **9954** | **Mean [years]** | **3152** | **276** | **11440** |
| SD | 128 | 57 | 340 | SD | 134 | 66 | 414 |
