# Supplementary material for: Hybrids as mirrors of the past: genomic footprints reveal spatio-temporal dynamics and extinction risk of alpine extremophytes in the mountains of Central Asia
Source: Front Plant Sci. 2024 Apr 17;15:1369732. doi: 10.3389/fpls.2024.1369732 (PMC11061500; doi:10.3389/fpls.2024.1369732)
Supplement: Supplementary Table 8 — Statistics of posterior distribution estimated for the model parameter r1 of Scenario 1 in DIYABC-RF analysis. [file Table_8.docx]

**Supplementary Table 8.** Estimations of posterior median as well as 5% and 95% quantiles (90% credibility interval) of the **parameter** **r_1_** (admixture rate derived from *Puccinellia pamirica* during interspecific hybridization events with *P. himalaica*) performed using 10,000 simulations of the best supported scenario (**Scenario 1**) based on ten replicate analyses. The parameter r_1_ was modeled using a prior distribution set between 0.01 and 0.99. The analysis was performed using the approximate Bayesian computation with supervised machine learning in DIYABC-RF ver. 1.2.1. Scenario 1 is shown on **Figure 6**.

| **North/South cluster model** | | | | **North/South population model** | | | |
| --- | --- | --- | --- | --- | --- | --- | --- |
| Reference table | Median | q5% | q95% | Reference table | Median | q5% | q95% |
| 1 | 0.508 | 0.455 | 0.571 | 1 | 0.522 | 0.424 | 0.609 |
| 2 | 0.508 | 0.455 | 0.567 | 2 | 0.514 | 0.420 | 0.606 |
| 3 | 0.510 | 0.458 | 0.558 | 3 | 0.522 | 0.428 | 0.611 |
| 4 | 0.510 | 0.458 | 0.557 | 4 | 0.521 | 0.418 | 0.607 |
| 5 | 0.510 | 0.454 | 0.564 | 5 | 0.517 | 0.419 | 0.612 |
| 6 | 0.513 | 0.455 | 0.567 | 6 | 0.518 | 0.428 | 0.612 |
| 7 | 0.510 | 0.456 | 0.566 | 7 | 0.517 | 0.418 | 0.607 |
| 8 | 0.510 | 0.456 | 0.560 | 8 | 0.516 | 0.421 | 0.607 |
| 9 | 0.512 | 0.456 | 0.568 | 9 | 0.519 | 0.419 | 0.607 |
| 10 | 0.509 | 0.458 | 0.566 | 10 | 0.514 | 0.420 | 0.604 |
| **Mean** | **0.510** | **0.456** | **0.564** | **Mean** | **0.518** | **0.421** | **0.608** |
| SD | **0.002** | **0.001** | **0.004** | SD | **0.003** | **0.004** | **0.003** |
