# Supplementary material for: Hybrids as mirrors of the past: genomic footprints reveal spatio-temporal dynamics and extinction risk of alpine extremophytes in the mountains of Central Asia
Source: Front Plant Sci. 2024 Apr 17;15:1369732. doi: 10.3389/fpls.2024.1369732 (PMC11061500; doi:10.3389/fpls.2024.1369732)
Supplement: Supplementary Table 9 — Statistics of posterior distribution estimated for the model parameter Scenario 1 in DIYABC-RF analysis. [file Table_9.docx]

**Supplementary Table 9.** Estimations of posterior median as well as 5% and 95% quantiles (90% credibility interval) of the **parameter** **t_2_** (time of the development of intraspecific genetic structure of *P. pamirica* before the occurrence of multiple post-local LGM interspecific hybridisation events with *P. himalaica*; t_2_ > t_1_) performed using 10,000 simulations of the best supported scenario (**Scenario 1**) based on ten replicate analyses. The parameter t_2_ was modelled using a prior distribution set between 10 and 25,000 generations (interpreted as a period after the local Last Glacial Maximum in the Pamir Mountains estimated between 50,000–100,000 years BP; we assumed 2-year generation time). The analysis was performed using the approximate Bayesian computation with supervised machine learning in DIYABC-RF ver. 1.2.1. Scenario 1 is shown on **Figure 6**.

| **North/South cluster model** | | | | **North/South population model** | | | |
| --- | --- | --- | --- | --- | --- | --- | --- |
| Reference table | Median | q5% | q95% | Reference table | Median | q5% | q95% |
| 1 | 11296 | 4579 | 20874 | 1 | 14604 | 6226 | 23662 |
| 2 | 11282 | 4036 | 21125 | 2 | 14724 | 6663 | 23344 |
| 3 | 11641 | 4461 | 20970 | 3 | 14032 | 5551 | 23604 |
| 4 | 10950 | 4198 | 21601 | 4 | 14375 | 6141 | 23176 |
| 5 | 11687 | 4579 | 21839 | 5 | 14432 | 6141 | 23358 |
| 6 | 10728 | 4289 | 20874 | 6 | 14681 | 6183 | 23809 |
| 7 | 11136 | 4416 | 20155 | 7 | 14868 | 6226 | 23728 |
| 8 | 11325 | 4460 | 22042 | 8 | 15378 | 6961 | 23713 |
| 9 | 11049 | 4575 | 20636 | 9 | 14442 | 6691 | 23701 |
| 10 | 11399 | 4575 | 20714 | 10 | 14680 | 5256 | 23803 |
| Mean [generations] | 11249 | 4417 | 21083 | Mean [generations] | 14622 | 6204 | 23590 |
| SD | 281 | 178 | 553 | SD | 336 | 485 | 208 |
| **Mean [years]** | **22498** | **8834** | **42166** | **Mean [years]** | **29243** | **12408** | **47180** |
| SD | 562 | 355 | 1106 | SD | 671 | 970 | 415 |
