# Supplementary material for: Hybrids as mirrors of the past: genomic footprints reveal spatio-temporal dynamics and extinction risk of alpine extremophytes in the mountains of Central Asia
Source: Front Plant Sci. 2024 Apr 17;15:1369732. doi: 10.3389/fpls.2024.1369732 (PMC11061500; doi:10.3389/fpls.2024.1369732)
Supplement: Supplementary Table 10 — Statistics of posterior distribution estimated for the model parameter t2 of Scenario 3 in DIYABC-RF analysis. [file Table_10.docx]

**Supplementary Table 10.** Estimations of posterior median as well as 5% and 95% quantiles (90% credibility interval) of the **parameter** **t_2_** (time of multiple post-local LGM interspecific hybridization events between *Puccinellia pamirica* and *P. himalaica*) performed using 10,000 simulations of the second best supported scenario (**Scenario 3**) based on ten replicate analyses. The parameter **t_2_** was modeled using a prior distribution set between 10 and 25,000 generations (interpreted as a period after the local Last Glacial Maximum in the Pamir Mountains estimated between 50,000–100,000 years BP; we assumed 2-year generation time). The analysis was performed using the approximate Bayesian computation with supervised machine learning in DIYABC-RF ver. 1.2.1. Scenario 3 is shown on **Figure 6**.

| **North/South cluster model** | | | | **North/South population model** | | | |
| --- | --- | --- | --- | --- | --- | --- | --- |
| Reference table | Median | q5% | q95% | Reference table | Median | q5% | q95% |
| 1 | 1555 | 115 | 6818 | 1 | 1372 | 51 | 6580 |
| 2 | 1475 | 95 | 6837 | 2 | 1565 | 94 | 7035 |
| 3 | 1391 | 79 | 5742 | 3 | 1525 | 95 | 6566 |
| 4 | 1537 | 128 | 6913 | 4 | 1412 | 110 | 6074 |
| 5 | 1332 | 92 | 6912 | 5 | 1650 | 92 | 6912 |
| 6 | 1565 | 91 | 6920 | 6 | 1409 | 126 | 6017 |
| 7 | 1409 | 109 | 6844 | 7 | 1359 | 125 | 6402 |
| 8 | 1565 | 84 | 6668 | 8 | 1555 | 143 | 6920 |
| 9 | 1295 | 95 | 5583 | 9 | 1259 | 125 | 6913 |
| 10 | 1259 | 110 | 6074 | 10 | 1399 | 110 | 6269 |
| Mean [generations] | 1438 | 100 | 6531 | Mean [generations] | 1450 | 107 | 6569 |
| SD | 111 | 15 | 496 | SD | 113 | 25 | 353 |
| **Mean [years]** | **2518** | **220** | **12149** | **Mean [years]** | **2901** | **214** | **13137** |
| SD | 2877 | 199 | 13062 | SD | 225 | 49 | 706 |
