# Supplementary material for: Hybrids as mirrors of the past: genomic footprints reveal spatio-temporal dynamics and extinction risk of alpine extremophytes in the mountains of Central Asia
Source: Front Plant Sci. 2024 Apr 17;15:1369732. doi: 10.3389/fpls.2024.1369732 (PMC11061500; doi:10.3389/fpls.2024.1369732)
Supplement: Supplementary Table 11 — Scenario choice with grouping in the North/South cluster model in DIYABC-RF analysis. [file Table_11.docx]

**Supplementary Table 11.** Lineage diversification history and scenarios on origins of *Puccinellia ×vachanica* emerging from *P. pamirica* and *P. himalaica*, tested by the approximate Bayesian computation with supervised machine learning in DIYABC-RF ver. 1.2.1. **Scenario choice** for each of the ten replicate analyses was based on 10 different reference tables comparing six tested scenarios in the **North/South cluster model** (118 individuals) – Scenario 1 was grouped with Scenario 3 for a scenario choice. For each reference table, the number of datasets simulated using DIYABC-RF was set to 10,000 per scenario and the number of RF-trees was 500. Scenarios are shown on Figure 3 in the main article. Scenarios are shown on **Figure 6**.

| **Reference table** | **Best scenario** | **Votes on scenario (proportion per 500 votes)** | | | | | **Prior error rate** | **Posterior probability (best scenario)** |
| --- | --- | --- | --- | --- | --- | --- | --- | --- |
|  |  |  |  |  |  |  |  |  |
|  |  | **1+3** | **2** | **4** | **5** | **6** |  |  |
| 1 | 1 | 0.608 | 0.042 | 0.222 | 0.018 | 0.110 | 0.197 | 0.781 |
| 2 | 1 | 0.622 | 0.028 | 0.204 | 0.020 | 0.132 | 0.198 | 0.831 |
| 3 | 1 | 0.614 | 0.022 | 0.204 | 0.020 | 0.132 | 0.198 | 0.874 |
| 4 | 1 | 0.600 | 0.020 | 0.236 | 0.018 | 0.112 | 0.198 | 0.862 |
| 5 | 1 | 0.620 | 0.034 | 0.226 | 0.012 | 0.128 | 0.198 | 0.834 |
| 6 | 1 | 0.610 | 0.020 | 0.238 | 0.030 | 0.092 | 0.197 | 0.840 |
| 7 | 1 | 0.618 | 0.022 | 0.202 | 0.030 | 0.136 | 0.197 | 0.800 |
| 8 | 1 | 0.638 | 0.024 | 0.224 | 0.028 | 0.106 | 0.197 | 0.865 |
| 9 | 1 | 0.654 | 0.022 | 0.206 | 0.020 | 0.114 | 0.197 | 0.849 |
| 10 | 1 | 0.620 | 0.030 | 0.190 | 0.020 | 0.106 | 0.197 | 0.834 |
| **Mean** | | **0.620** | **0.026** | **0.215** | **0.022** | **0.117** | **0.197** | **0.837** |
| **SD** | | 0.015 | 0.007 | 0.015 | 0.006 | 0.014 | 0.001 | 0.027 |
