# Supplementary material for: Hybrids as mirrors of the past: genomic footprints reveal spatio-temporal dynamics and extinction risk of alpine extremophytes in the mountains of Central Asia
Source: Front Plant Sci. 2024 Apr 17;15:1369732. doi: 10.3389/fpls.2024.1369732 (PMC11061500; doi:10.3389/fpls.2024.1369732)
Supplement: Supplementary Table 12 — Scenario choice with grouping in the North/South population model in DIYABC-RF analysis. [file Table_12.docx]

**Supplementary Table 12.** Lineage diversification history and scenarios on origins of *Puccinellia ×vachanica* emerging from *P. pamirica* and *P. himalaica*, tested by the approximate Bayesian computation with supervised machine learning in DIYABC-RF ver. 1.2.1. **Scenario choice** for each of the ten replicate analyses was based on 10 different reference tables comparing six tested scenarios in the **North/South population model** (27 individuals) – Scenario 1 was grouped with Scenario 3 for a scenario choice. For each reference table, the number of datasets simulated using DIYABC-RF was set to 10,000 per scenario and the number of RF-trees was 500. Scenarios are shown on **Figure 6**.

| **Reference table** | **Best scenario** | **Votes on scenario (proportion per 500 votes)** | | | | | **Prior error rate** | **Posterior probability (best scenario)** |
| --- | --- | --- | --- | --- | --- | --- | --- | --- |
|  |  |  |  |  |  |  |  |  |
|  |  | **1+3** | **2** | **4** | **5** | **6** |  |  |
| 1 | 1 | 0.838 | 0.022 | 0.116 | 0.008 | 0.016 | 0.233 | 0.881 |
| 2 | 1 | 0.844 | 0.012 | 0.126 | 0.000 | 0.018 | 0.233 | 0.875 |
| 3 | 1 | 0.828 | 0.018 | 0.124 | 0.004 | 0.026 | 0.234 | 0.874 |
| 4 | 1 | 0.868 | 0.012 | 0.104 | 0.004 | 0.012 | 0.232 | 0.896 |
| 5 | 1 | 0.840 | 0.014 | 0.122 | 0.004 | 0.020 | 0.232 | 0.902 |
| 6 | 1 | 0.860 | 0.012 | 0.110 | 0.004 | 0.014 | 0.232 | 0.895 |
| 7 | 1 | 0.830 | 0.018 | 0.128 | 0.004 | 0.020 | 0.232 | 0.894 |
| 8 | 1 | 0.828 | 0.016 | 0.140 | 0.002 | 0.014 | 0.232 | 0.914 |
| 9 | 1 | 0.830 | 0.014 | 0.136 | 0.004 | 0.016 | 0.233 | 0.887 |
| 10 | 1 | 0.858 | 0.016 | 0.106 | 0.004 | 0.016 | 0.232 | 0.898 |
| **Mean** | | **0.842** | **0.015** | **0.121** | **0.004** | **0.017** | **0.233** | **0.892** |
| **SD** | | 0.014 | 0.003 | 0.012 | 0.002 | 0.004 | 0.001 | 0.012 |
