# Supplementary material for: Hybrids as mirrors of the past: genomic footprints reveal spatio-temporal dynamics and extinction risk of alpine extremophytes in the mountains of Central Asia
Source: Front Plant Sci. 2024 Apr 17;15:1369732. doi: 10.3389/fpls.2024.1369732 (PMC11061500; doi:10.3389/fpls.2024.1369732)
Supplement: Supplementary Presentation 1 — SNP Marker discovery and genotyping using the DArTseq platform. [file Presentation_1.pdf]

Supplementary Material 1

SNP Marker Discovery and Genotyping via the DArTseq Platform

Genomic DNA was extracted from dried leaf tissues using the Genomic Mini AX Plant Kit (A&A Biotechnology, Poland). Quality control was performed using the NanoDrop One (Thermo Scientific, USA) and agarose gel electrophoresis visualisation. The concentration of each sample was adjusted to 95 ng/µL. Materials were sent to the Diversity Arrays Technology Pty Ltd (Canberra, Australia) for sequencing and marker identification.

For *Puccinellia* specimens, the PstI-MseI method was selected. DNA samples were processed in digestion/ligation reactions principally as per Kilian et al. (2012) but replacing a single PstI-compatible adaptor with two different adaptors corresponding to two different Restriction Enzyme (RE) overhangs. The PstI-compatible adapter was designed to include Illumina flowcell attachment sequence, sequencing primer sequence and staggered, varying length barcode region, similar to the sequence reported by Elshire et al. (2011). Reverse adapter contained flowcell attachment region and MseI-compatible overhang sequence. Only mixed fragments (PstI-MseI) were effectively amplified in 30 rounds of PCR using the following reaction conditions: 1) 94°C for 1 min, 2) 30 cycles of: 94°C for 20 sec, 58°C for 30 sec, and 72°C for 45 sec, 3) 72°C for 7 min. After PCR, equimolar amounts of amplification products from each sample of the 96-well microtiter plate were bulked and applied to c-Bot (Illumina) bridge PCR followed by sequencing on Illumina Novaseq6000. The sequencing (single read) was run for 83 cycles.

Sequences generated from each lane were processed using the proprietary DArT analytical pipelines. In the primary pipeline, the fastq files were first processed to filter away poor quality sequences, applying more stringent selection criteria to the barcode region compared to the rest of the sequence. In that way the assignments of the sequences to specific samples carried in the barcode split step were reliable. Approximately 3,300,000 sequences per sample were identified (CoV of 7%) and used in the marker calling. Finally, identical sequences were collapsed into fastqcoll files. The fastqcoll files were groomed using the DArT PLs proprietary algorithm which corrects low quality base from singleton tag into a correct base using collapsed tags with multiple members as a template. The groomed fastqcoll files were used in the secondary pipeline for the DArT PLs proprietary SNP calling algorithms (DArTsoft14). For the SNP calling, all tags from all libraries included in the DArTsoft14 analysis are clustered using the DArT PLs C++ algorithm at the threshold distance of 3, followed by parsing of the clusters into separate SNP loci using a range of

technical parameters, especially the balance of read counts for the allelic pairs. Additional selection criteria were added to the algorithm based on analysis of approximately 1,000 controlled cross populations. Test for Mendelian distribution of alleles in these populations facilitated selection of technical parameters, discriminating well true allelic variants from paralogous sequences. In addition, multiple samples were processed from DNA to allelic calls as technical replicates and scoring consistency was used as the main selection criteria for high quality/low error rate markers. Calling quality was assured by high average read depth per locus (average across all markers was around 30 reads/locus). The average number of sequences per sample in this analysis was 3.29 million and the average number of unique sequences per sample was 480,000. For 48 samples technical replicates (libraries) were processed to obtain estimates for marker calling reproducibility.

References

Elshire R.J., Glaubitz J.C., Sun Q., Poland J.A., Kawamoto K., Buckler E.S., Mitchell S.E. 2011. A Robust, Simple Genotyping-by-Sequencing (GBS) Approach for High Diversity Species. PLoS One. 6:e19379.

Kilian A., Wenzl P., Huttner E., Carling J., Xia L., Blois H., Caig V., Heller-Uszynska K., Jaccoud D., Hopper C., Aschenbrenner-Kilian M., Evers M., Peng K., Cayla C., Hok P., Uszynski G. 2012. Diversity arrays technology: a generic genome profiling technology on open platforms. Methods Mol. Biol. 888:67–89.

Acknowledgements

We would like to thank Andrzej Kilian (Diversity Arrays Technology Pty Ltd, Canberra, Australia) for providing us with a detailed description of the procedure underlying SNP marker discovery for our model system.
